# Creation of new germplasm resources, development of SSR markers, and screening of monoterpene synthases in thyme

**DOI:** 10.1186/s12870-022-04029-2

**Published:** 2023-01-06

**Authors:** Meiyu Sun, Li Zhu, Yanan Zhang, Ningning Liu, Jinzheng Zhang, Hui Li, Hongtong Bai, Lei Shi

**Affiliations:** 1 Key Laboratory of Plant Resources, Institute of Botany, Chinese Academy of Sciences, Beijing, 100093 China; 2 China National Botanical Garden, Beijing, 100093 China; 3 University of Chinese Academy of Sciences, Beijing, 100049 China

**Keywords:** Thyme, Essential oil, Hybrid, SSR marker, Monoterpene synthase

## Abstract

**Background:**

Thyme derived essential oil and its components have numerous applications in pharmaceutical, food, and cosmetic industries, owing to their antibacterial, antifungal, and antiviral properties. To obtain thyme essential oil with different terpene composition, we developed new germplasm resources using the conventional hybridization approach.

**Results:**

Phenotypic characteristics, including essential oil yield and composition, glandular trichome density, plant type, and fertility, of three wild Chinese and seven European thyme species were evaluated. Male-sterile and male-fertile thyme species were crossed in different combinations, and two F_1_ populations derived from *Thymus longicaulis* (Tl) × *T. vulgaris* ‘Fragrantissimus’ (Tvf) and *T. vulgaris* ‘Elsbeth’ (Tve) × *T. quinquecostatus* (Tq) crosses were selected, with essential oil yield and terpene content as the main breeding goals. Simultaneously, simple sequence repeat (SSR) primers were developed based on the whole-genome sequence of *T. quinquecostatus* to authenticate the F_1_ hybrids. A total of 300 primer pairs were selected, and polymerase chain reaction (PCR) was carried out on the parents of the two hybrid populations (Tl, Tvf, Tve, and Tq). Based on the chemotype of the parents and their F_1_ progenies, we examined the expression of genes encoding two γ-terpinene synthases, one α-terpineol synthase, and maybe one geraniol synthase in all genotypes by quantitative real-time PCR (qRT-PCR).

**Conclusion:**

We used hybridization to create new germplasm resources of thyme, developed SSR markers based on the whole-genome sequence of *T. quinquecostatus*, and screened the expression of monoterpene synthase genes in thyme. The results of this study provide a strong foundation for the creation of new germplasm resources, construction of the genetic linkage maps, and identification of quantitative trait loci (QTLs), and help gain insight into the mechanism of monoterpenoids biosynthesis in thyme.

**Supplementary Information:**

The online version contains supplementary material available at 10.1186/s12870-022-04029-2.

## Background

Species belonging to the genus *Thymus* (family Lamiaceae) are commonly used for food, cosmetic, and medicinal purposes [1–3]. Thyme (*Thymus*) species are important aromatic and medicinal plants that have been used as traditional medicine for thousands of years in the Mediterranean basin [4]. These species are highly appreciated for the wide spectrum of pharmacological properties of their essential oils. The antirheumatic, antiseptic, antispasmodic, antimicrobial, anti-inflammatory, carminative, diuretic, and expectorant activities of thyme essential oil have been researched and validated [5–8]. The main components of thyme essential oil belong to the chemical classes of terpenoids, terpene alcohols, phenolic derivatives, ketones, aldehydes, ethers, and esters [9]. Generally, these oils contain oxygenated monoterpenoids (e.g., thymol, carvacrol, γ-terpinene, *p*-cymene, 1,8-cineole, linalool, α-terpineol, geraniol, and borneol), sesquiterpenoids (e.g., β-caryophyllene), and oxygenated sesquiterpenoids (e.g., caryophyllene oxide) [4, 8, 9]. Depending on the characteristics and composition of thyme essential oil, the occurrence of different chemotypes have been described within several species of *Thymus* [2, 9–11], including *T. vulgaris*.

To date, a few studies have been conducted by scientific research institutions and companies on thyme breeding, especially in European countries, and few of the high-performance industrial and horticultural varieties developed in these studies have been cultivated. In the 4th International Symposium on the Breeding of Medicinal Aromatic Plants, a breeding study was reported, in which male-sterile and male-fertile clones were crossed to optimize the terpenoid content and yield of thyme (*T. vulgaris*) [12]. In this study, 56 new hybrids, which were tested by assessing the homogeneity, dry weight, essential oil yield, winter frost tolerance, and seed production potential of the parents, were obtained from 2000 to 2002. The most dominant hybrid, named ‘Varico 3’, showed 4.9% essential oil yield and thymol-type chemotype [12]. Such hybrid varieties have been entered into the market. Clone T-12, with high phenol content, was selected from among 10 *T. vulgaris* clones [13]. The global collection of *Thymus* resources is highly diverse, with more than 300 species, which are native to the Mediterranean basin and are widely distributed in the temperate regions of Europe, North Africa, and Asia [14]. In China, *Thymus* species are mainly distributed in the northwest, north, and northeast regions based on the Flora of China [15]. Therefore, according to the breeding goals, wild Chinese thyme species could be crossed with European thyme species to develop a series of new varieties for applications in different fields.

With the rapid development of modern molecular biology approaches and genome sequencing technologies, DNA-based molecular markers have become an important tool for cultivar identification, fingerprinting [16, 17], and genetic diversity analysis [18–20]. To improve the industrial applications of medicinal and aromatic plants, breeders often select plants with high genetic divergence and essential oil content [17]. Phenotypic variation could be very valuable for molecular breeding approaches such as marker-assisted selection (MAS), which has been very helpful in elucidating the genetic diversity of plant species. A dendrogram, based on cluster analysis, showed that *T. daenensis* and *T. fallax* are clearly distinct from the other *Thymus* species, indicating that *T. daenensis* shares some genetic similarity with *T. fallax* [21]. Previously, several studies used randomly amplified polymorphic DNA (RAPD) markers to investigate the genetic diversity and essential oil composition of various *Thymus* species as well as the phylogenetic relationship among these species [11, 17, 22].

Phenolic monoterpenoids in thyme are synthesized via two pathways: the mevalonate (MVA) pathway and 2-C-methyl-D-erythritol-4-phosphate (MEP) pathway [23]. Isopentenyl diphosphate (IPP) and dimethylallyl diphosphate (DMAPP) are canonically condensed head-to-tail by trans-prenyltransferases to generate geranyl diphosphate (GPP) and farnesyl diphosphate (FPP). Terpene synthase (TPS) converts GPP and FPP into the basic skeleton of monoterpenes (C10) and sesquiterpenes (C15), respectively. TPSs have been characterized as multifunctional enzymes, owing to multiple single amino acid substitutions, which result in altered metabolic profiles [24–26]. A wide range of TPSs and other terpene-modifying enzymes have been characterized to date [27]. *TPS* genes have been identified in several *Thymus* species, including *T. caespititius* [28–30], *T. vulgaris* [31–34], *T. serpyllum* [31], *T. albicans* [35], and *T. citriodorus*.

In this study, male-sterile and male-fertile thyme varieties were crossed in two different combinations, *T. longicaulis* (Tl) × *T. vulgaris* ‘Fragrantissimus’ (Tvf) and *T. vulgaris* ‘Elsbeth’ (Tve) × *T. quinquecostatus* (Tq), to generate two F_1_ populations. Then, simple sequence repeat (SSR) markers were developed based on the whole-genome sequence of *T. quinquecostatus* [36], to authenticate all F_1_ individuals. In addition, the expression profiles of two γ-terpinene synthase genes (*Tq13G005250.1* and *Tq02G002290.1*), one geraniol synthase gene (*Tq04G005190.1*), and maybe one α-terpineol synthase gene (*Tq03G001560.1*) were analyzed by quantitative real-time PCR (qRT-PCR). Overall, this study provides a valuable collection of new thyme varieties, which could be used for MAS and the verification of *TPS* gene function in future studies.

## Results

### Phenotypic evaluation of different thyme species and construction of F_1_ hybrid populations

**Fig. 1:**
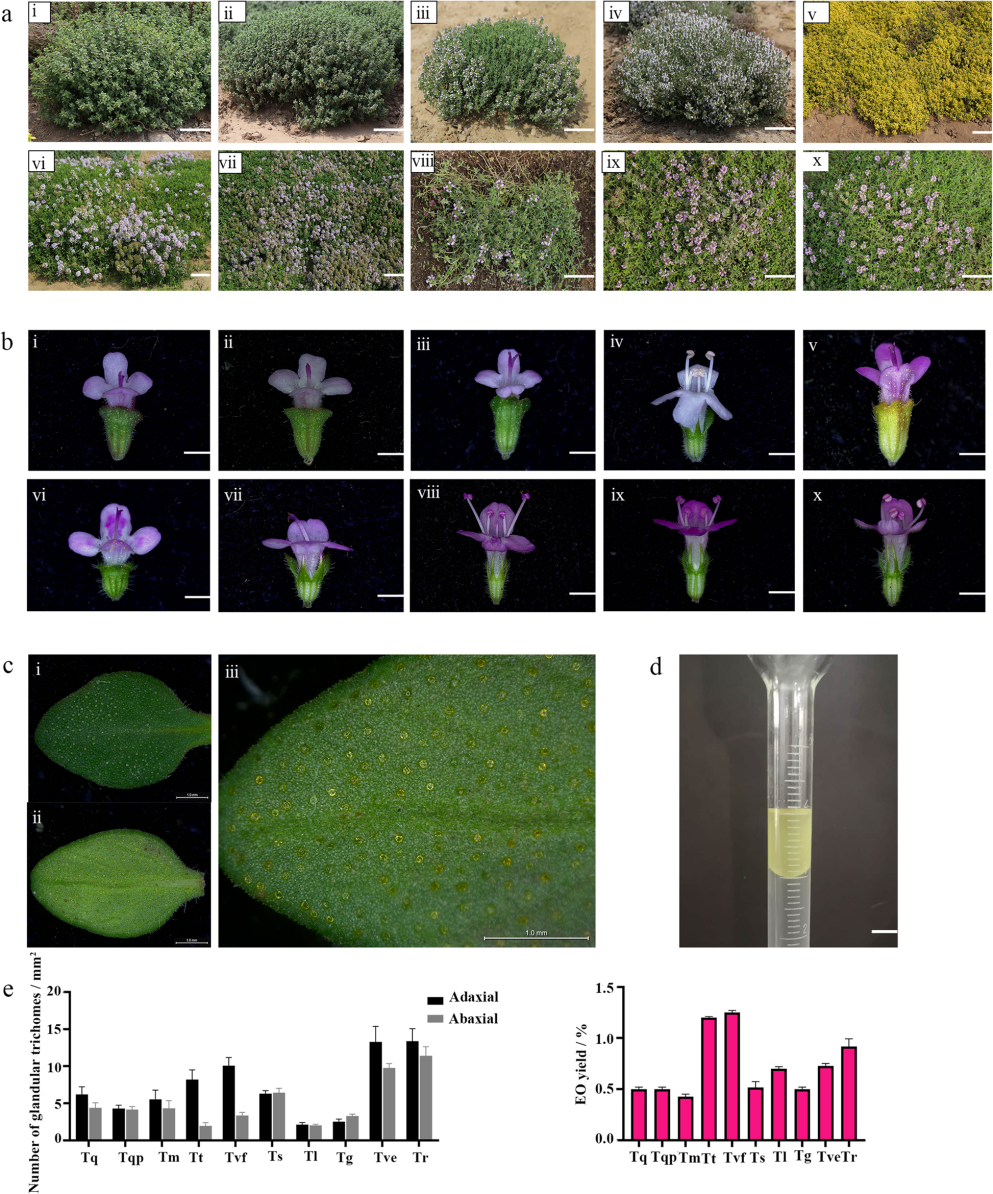
Phenotypic evaluation of three wild Chinese and seven European thyme (*Thymus*) species. **a** Plant types. **i** *T. rotundifolius* (Tr); **ii** *T. vulgaris* ‘Elsbeth’ (Tve); **iii** *T. thracicus* (Tt); **iv** *T. vulgaris* ‘Fragrantissimus’ (Tvf); **v** *T. serpyllum* ‘Aureus’ (Ts); **vi** *T. guberlinesis* (Tg); **vii** *T. longicaulis* (Tl); **viii** *T. quinquecostatus* (Tq); **ix** *T. quinquecostatus* var. *przewalskii* (Tqp); **x** *T. mongolicus* (Tm). **b** Floral organ arrangement. **i** *T. rotundifolius* (Tr); **ii** *T. vulgaris* ‘Elsbeth’ (Tve); **iii** *T. thracicus* (Tt); **iv** *T. vulgaris* ‘Fragrantissimus’ (Tvf); **v** *T. serpyllum* ‘Aureus’ (Ts); **vi** *T. guberlinesis* (Tg); **vii** *T. longicaulis* (Tl); **viii** *T. quinquecostatus* (Tq); **ix** *T. quinquecostatus* var. *przewalskii* (Tqp); **x** *T. mongolicus* (Tm). Scale bars = 1 mm. **c** Glandular trichomes on the adaxial and abaxial surface of leaves. **i** Adaxial; **ii** Abaxial; **iii** Glandular trichomes on the abaxial surface. **d** Images of thyme essential oil. **e** Glandular trichome number on the adaxial and abaxial surfaces of leaves. **f** Essential oil (EO) yield

Thyme is a herbaceous perennial or sub-shrub with valuable medicinal and aromatic properties. Thyme plants are of two types, depending on their growth habit: erect-type and creeping-type. These two plant types display remarkable differences in morphology. The plant types of 10 different thyme species are shown in Fig. 1a; Table 1. Among these species, *T. rotundifolius* (Tr), *T. vulgaris* ‘Elsbeth’ (Tve), *T. thracicus* (Tt), and *T. vulgaris* ‘Fragrantissimus’ (Tvf) are erect-type, and *T. serpyllum* ‘Aureus’ (Ts), *T. guberlinesis* (Tg), *T. longicaulis* (Tl), *T. quinquecostatus* (Tq), *T. quinquecostatus* var. *przewalskii* (Tqp), and *T. mongolicus* (Tm) are creeping-type. The fertility of different thyme species is shown in Fig. 1b. Tr, Tve, Tg, Tt, Ts, and Tl contained only stigma and no pollen (stamen), and were therefore male-sterile. By contrast, Tq, Tqp, Tm, and Tvf possessed both stigma and pollen (stamen), and therefore were categorized as male-fertile (Table 1). The increased genetic diversity of thyme species could be attributed partially to the consistent introgression of wild Chinese thyme germplasm into the male-sterile and erect-type European thyme germplasm during long cultivation periods, and partially to the adaptation of thyme species to new environments in new geographical locations.

**Table 1:** Phenotypic characteristics of three wild Chinese and seven European thyme species

Species	Chemotype	Plant type	Oil yield (%)	Pollen	Seed	Sterility phenotype	Flowering phase
*Thymus quinquecostatus*	Carvacrol	Creeping	0.50	Present	Present	Male-fertile	2020.4.20–2020.8.30
*Thymus quinquecostatus* var. *przewalskii*	Carvacrol	Creeping	0.50	Present	Present	Male-fertile	2020.4.20–2020.8.30
*Thymus mongolicus*	Thymol	Creeping	0.40	Present	Present	Male-fertile	2020.5.12–2020.8.30
*Thymus rotundifolius*	Thymol	Erect	0.95	Absent	Absent	Male-sterile	2020.6.20–2020.7.30
*Thymus vulgaris* ‘Elsbeth’	Thymol	Erect	0.75	Absent	Absent	Male-sterile	2020.6.1–2020.6.20
*Thymus guberlinesis*	Thymol	Creeping	0.50	Absent	Absent	Male-sterile	2020.5.6–2020.5.20
*Thymus thracicus*	Thymol	Erect	1.20	Absent	Absent	Male-sterile	2020.5.8–2020.5.30
*Thymus serpyllum* ‘Aureus’	Thymol	Creeping	0.52	Absent	Absent	Male-sterile	2020.5.22–2020.6.15
*Thymus longicaulis*	Geraniol	Creeping	0.70	Absent	Absent	Male-sterile	2020.4.20–2020.5.15
*Thymus vulgaris* ‘Fragrantissimus’	α–terpineol	Erect	1.25	Present	Present	Male-fertile	2020.4.20–2020.5.10

Glandular trichomes are specialized hairs that originate from the epidermal cells of flowers, leaves, and stems. These organs exist as two types, peltate and capitate, on the surface of approximately 30% of all vascular plants, including lavender, thyme, rosemary, oregano, basil, and other Lamiaceae species [37]. Glandular trichomes are responsible for a significant portion of the secondary metabolite of a plant [38], and serve as the storage and synthesis sites of terpenoids [39]. The regulation of glandular trichome formation related genes potentially underlies the regulation of glandular trichome density (number of glandular trichomes per unit area) for increasing the terpenoid content of plants.

Tve and Tr showed the highest glandular trichome density on both the adaxial and abaxial leaf surfaces (Fig. 1c, e). Glandular trichome density on the adaxial leaf surface was the second highest in Tvf and Tt. The glandular trichome density of leaves, overall, decreased in the following order: Tve, Tr, Tvf, Tt, and Ts. The essential oil yield of thyme species is shown in Fig. 1d, f and Supplementary Fig. S1. Tvf showed the highest essential oil yield (1.25 mL**·**100 g^− 1^), followed by Tt (1.20 mL**·**100 g^− 1^), Tr (0.95 mL**·**100 g^− 1^), Tve (0.75 mL**·**100 g^− 1^), and Tl (0.70 mL**·**100 g^− 1^). The essential oil yield of Tq, Tqp, Tm, Tg, and Ts varied between 0.40 mL**·**100 g^− 1^ and 0.50 mL**·**100 g^− 1^ (Table 1; Supplementary Fig. S1). There was a certain correlation between essential oil yield and glandular trichome density, the higher the density of glandular trichome, the higher the yield of essential oil (Fig. 1e, f).

**Fig. 2:**
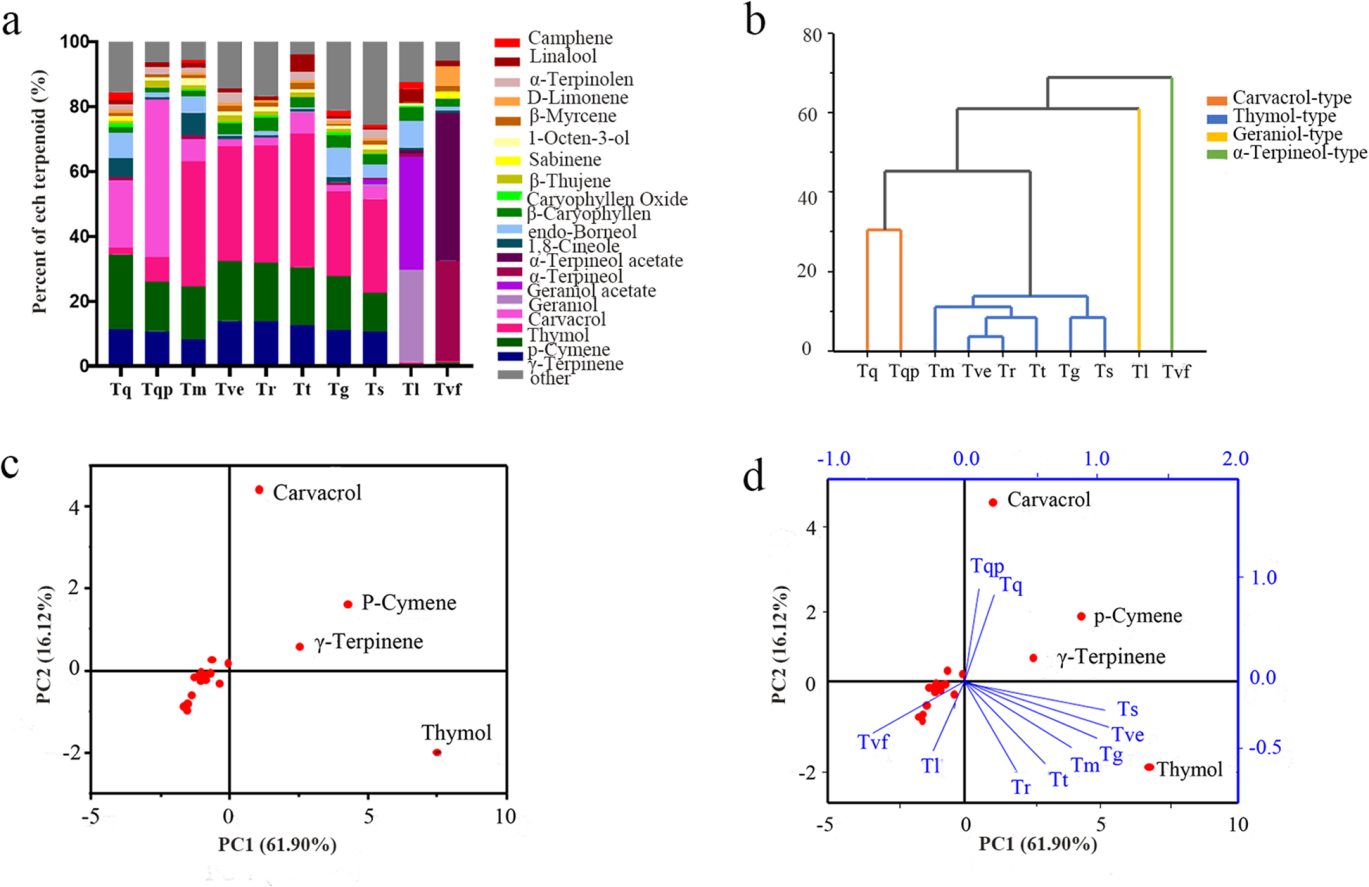
Relative content, cluster analysis, and principal component analysis (PCA) analysis of volatile organic compounds (VOCs) in the essential oil of 10 thyme species. **a** Histogram of the relative content of terpenoids. **b** Cluster analysis of 10 thyme species, based on their essential oil composition. **c** Loading plot of active terpenoids. **d** Score plot of essential oil compositions

The relative contents of essential oil components in 10 different thyme species are shown in Fig. 2a. Only 20 compounds showed relative contents of > 0.3% and were shared by 10 different thyme species (Table 2). The most abundant volatile compounds in Tq essential oil were *p*-cymene and carvacrol, which accounted for 23.00% and 20.74% (relative content), respectively. Tqp showed the highest content of carvacrol, which accounted for 48.37% of the total content of volatile compounds. In Tm, thymol was the most abundant volatile compound (38.57%), followed by *p*-cymene (16.40%). Similarly, in Tve, thymol was the most abundant volatile compound (35.43%), followed by *p*-cymene (18.42%), and γ-terpinene (13.96%). The thymol contents of Tr, Tt, Tg, and Ts were 36.02%, 41.04%, 26.26%, and 28.96%, respectively. In Tl, geranyl acetate (34.81%) was the most predominant volatile compounds, followed by geraniol (28.54%). The most abundant volatile compounds in Tvf were α-terpineol acetate (45.46%) and α-terpineol (30.84%).

**Table 2:** Relative contents of volatile terpenoids in the essential oil of 10 thyme species

No.	Terpenoid	RI Cal^a^	RI Lit^b^	Relative content (%)^c^									
				Tq	Tqp	Tm	Tve	Tr	Tt	Tg	Ts	Tl	Tvf
**1**	γ-Terpinene	1,059	1,060	11.45 ± 0.07c	10.77 ± 0.09e	8.19 ± 0.02 g	13.96 ± 0.08a	13.90 ± 0.09a	12.61 ± 0.08b	11.18 ± 0.13d	10.59 ± 0.05f	0.70 ± 0.02 h	0.48 ± 0.01i
**2**	*p-*Cymene	1,026	1,025	23.00 ± 0.09a	15.34 ± 0.19e	16.40 ± 0.15d	18.42 ± 0.12b	18.08 ± 0.17c	17.98 ± 0.05c	16.50 ± 0.33d	12.03 ± 0.1f	-	0.62 ± 0.01 g
**3**	Thymol	1,292	1,291	2.12 ± 0.05 h	7.59 ± 0.12 g	38.57 ± 0.25b	35.43 ± 0.14d	36.02 ± 0.14c	41.04 ± 0.13a	26.26 ± 0.05f	28.96 ± 0.09e	0.57 ± 0.01i	0.59 ± 0.01i
**4**	Carvacrol	1,304	1,299	20.74 ± 0.07b	48.37 ± 0.23a	6.87 ± 0.06c	2.27 ± 0.05 g	2.48 ± 0.03f	6.46 ± 0.06d	1.95 ± 0.03 h	3.78 ± 0.02e	-	-
**5**	Geraniol	1,259	1,255	-	-	-	-	-	0.49 ± 0.01c	-	0.69 ± 0.02b	28.54 ± 0.20a	-
**6**	Geraniol acetate	1,388	1,382	-	-	-	-	-	-	-	1.50 ± 0.01b	34.81 ± 0.22a	-
**7**	α-Terpineol	1,193	1,189	0.95 ± 0.01c	-	1.11 ± 0.01b	-	-	-	0.75 ± 0.01d	0.50 ± 0.01e	0.85 ± 0.02 cd	30.84 ± 0.24a
**8**	α-Terpineol acetate	1,355	1,350	-	-	-	-	-	-	-	-	1.17 ± 0.02b	45.46 ± 0.10a
**9**	Eucalyptol	1,030	1,032	5.78 ± 0.01b	0.93 ± 0.02d	7.02 ± 0.07a	0.81 ± 0.02ef	0.84 ± 0.02e	0.64 ± 0.01 g	1.71 ± 0.03c	-	0.77 ± 0.02f	0.67 ± 0.01 g
**10**	endo-Borneol	1,165	1,167	7.89 ± 0.06c	1.37 ± 0.05f	4.96 ± 0.05d	0.55 ± 0.01 h	1.23 ± 0.01 g	0.55 ± 0.01 h	9.02 ± 0.05a	4.13 ± 0.02e	8.18 ± 0.06b	1.26 ± 0.03 g
**11**	β-Caryophyllen	1,421	1,419	1.74 ± 0.01 g	1.56 ± 0.03 h	1.75 ± 0.02 g	3.37 ± 0.04d	4.01 ± 0.03b	3.11 ± 0.03e	3.79 ± 0.07c	3.07 ± 0.02e	4.19 ± 0.03a	2.60 ± 0.01f
**12**	Caryophyllen oxide	1,585	1,581	1.14 ± 0.01a	-	0.51 ± 0.01d	0.47 ± 0.01e	0.74 ± 0.02c	-	0.93 ± 0.02b	0.34 ± 0.01f	0.48 ± 002e	-
**13**	α-Thujene	926	929	0.91 ± 0.01 h	1.96 ± 0.05b	1.21 ± 0.01f	2.09 ± 0.02a	1.27 ± 0.01e	1.53 ± 0.02c	1.07 ± 0.02 g	1.30 ± 0.01d	-	-
**14**	Sabinene	972	974	0.81 ± 0.01b	-	-	-	-	-	-	-	-	2.18 ± 0.05a
**15**	1-Octen-3-ol	978	980	0.64 ± 0.01i	1.12 ± 0.03e	2.07 ± 0.03a	1.20 ± 0.03d	1.30 ± 0.02c	0.92 ± 0.01f	0.88 ± 0.01 g	1.39 ± 0.02b	0.69 ± 0.02 h	-
**16**	β-Myrcene	991	991	0.96 ± 0.01 g	1.09 ± 0.02f	1.14 ± 0.02e	1.73 ± 0.01b	1.37 ± 0.01d	1.86 ± 0.01a	0.51 ± 0.01 h	1.12 ± 0.01e	0.39 ± 0.03i	1.62 ± 0.03c
**17**	D-Limonene	1,028	1,032	0.89 ± 0.01c	-	0.70 ± 0.01e	0.90 ± 0.03c	0.80 ± 0.02d	0.81 ± 0.01d	1.09 ± 0.02b	0.77 ± 0.02d	-	6.04 ± 0.06a
**18**	α-Terpinolen	1,087	1,088	1.65 ± 0.02 g	2.10 ± 0.04f	1.58 ± 0.02 h	3.24 ± 0.01b	2.52 ± 0.02e	2.76 ± 0.03d	3.98 ± 0.02a	2.83 ± 0.01c	-	-
**19**	Linalool	1,099	1,099	1.17 ± 0.03f	1.52 ± 0.03d	1.33 ± 0.02e	1.06 ± 0.02 g	1.21 ± 0.02f	5.33 ± 0.02a	0.72 ± 0.02 h	0.38 ± 0.01i	4.12 ± 0.05b	1.81 ± 0.02c
**20**	Camphene	949	952	2.60 ± 0.01a	-	0.92 ± 0.01d	-	-	-	1.84 ± 0.04c	-	2.15 ± 0.04b	-

^a^ RI Cal, calculated according to C7–C40

^b^ RI Lit, obtained by searching the mass spectrum database NIST v14.0

^c^ Tq, *T. quinquecostatus*; Tqp, *T. quinquecostatus* var. *przewalskii*; Tm, *T. mongolicus*; Tve, *T. vulgaris* ‘Elsbeth’; Tr, *T. rotundifolius*; Tt, *T. thracicus*; Tg, *T. guberlinesis*; Ts, *T. serpyllum* ‘Aureus’; Tl, *T. longicaulis*; Tvf, *T. vulgaris* ‘Fragrantissimus’

Cluster analysis of the 20 main compounds found in the essential oil of 10 different thyme species (Fig. 2b) revealed four clusters. Tq and Tqp, which contained carvacrol as the most abundant compound (carvacrol-type essential oil), with relative contents of 20.74% and 48.37%, respectively, clustered together; Tm, Tve, Tr, Tt, Tg, and Ts grouped together, and contained thymol as the most abundant compound (thymol-type essential oil); and Tl and Tvf clustered separately (geraniol-type and α-terpineol-type essential oil, respectively). Principal component analysis (PCA) analysis was carried out on the main compounds found in the essential oils of all 10 thyme species (Fig. 2c, d). The results showed that the relatively high contents of thymol, carvacrol, *p*-cymene, and γ-terpinene contributed greatly to the volatile components of the essential oil of all 10 species. Tq and Tqp grouped together in the first quadrant, and their corresponding characteristic volatile substances included carvacrol, *p*-cymene, and γ-terpinene; Tvf and Tl were distributed in the third quadrant, which corresponded to volatiles geraniol and α-terpineol; Tm, Tve, Tr, Tt, Tg, and Ts were distributed in the fourth quadrant, and the corresponding predominant volatile was thymol (Fig. 2d); these results verified the results of cluster analysis.

### Development and application of SSR markers

The chromosome-level genome assembly and annotation using high-fidelity (HiFi) and chromatin conformation capture (Hi-C) technologies revealed 13 chromosomes in *T. quinquecostatus*, with a total length of 528.66 Mb, 70.61% (373.28 Mb) of which was annotated as highly repetitive [36]. A total of 239,400 tandem repeats were identified, accounting for 48.30 Mb (9.14%) of the genome. Additionally, 191,847 SSR loci were detected, of which 183,536 (95.67%) could be used for primer design. Among the top 10 contigs with the greatest distribution of SSR sites, Contig00377 showed the highest number of SSR sites (4,774), and Contig00721 contained the least number of SSR sites (2,089) (Supplementary Fig. S2a). The density of SSR loci (number of SSR loci per unit Mb), among these 10 contigs, was the highest in Contig00808 (435) and lowest in Contig00630 (264) (Supplementary Fig. S2b). Seven dinucleotide, twelve trinucleotide, and one tetranucleotide repeats were detected among the SSR loci of *T. quinquecostatus* (Supplementary Fig. S2c). Among the dinucleotide repeats, the CT/AG-type repeat was the most abundant (23.70%), followed by TC/GA (20.50%), TA/TA (14.80%), AT/AT (12.00%), TG/CA (3.80%), GT/AC (3.60%), and GC/GC (0.40%). Among the trinucleotide repeats, ATT/AAT accounted for the highest proportion (2.50%), followed by TTA/TAA (2.00%), TTC/GAA (1.60%), ATA/TAT (1.60%), AGA/TCT (1.50%), CTT/AAG (1.20%), GCC/GGC (0.60%), CCG/CGG (0.50%), GAG/CTC (0.50%), CGC/GCG (0.40%), ATC/GAT (0.40%), and GGA/TCC (0.30%). AAAT/ATTT (0.40%) was the only tetranucleotide repeat type identified in the *T. quinquecostatus* genome (Supplementary Fig. S2c). The length of SSR loci ranged from 18 to 87 bp in the *T. quinquecostatus* genome, with 10 bp SSRs being the most abundant (61,865, accounting for 32.00% of all SSR loci) and 25 bp SSR loci being the least abundant (1,356, 0.71%). The number of SSRs gradually decreased with the increase in repeat length (Supplementary Fig. S2d).

**Fig. 3:**
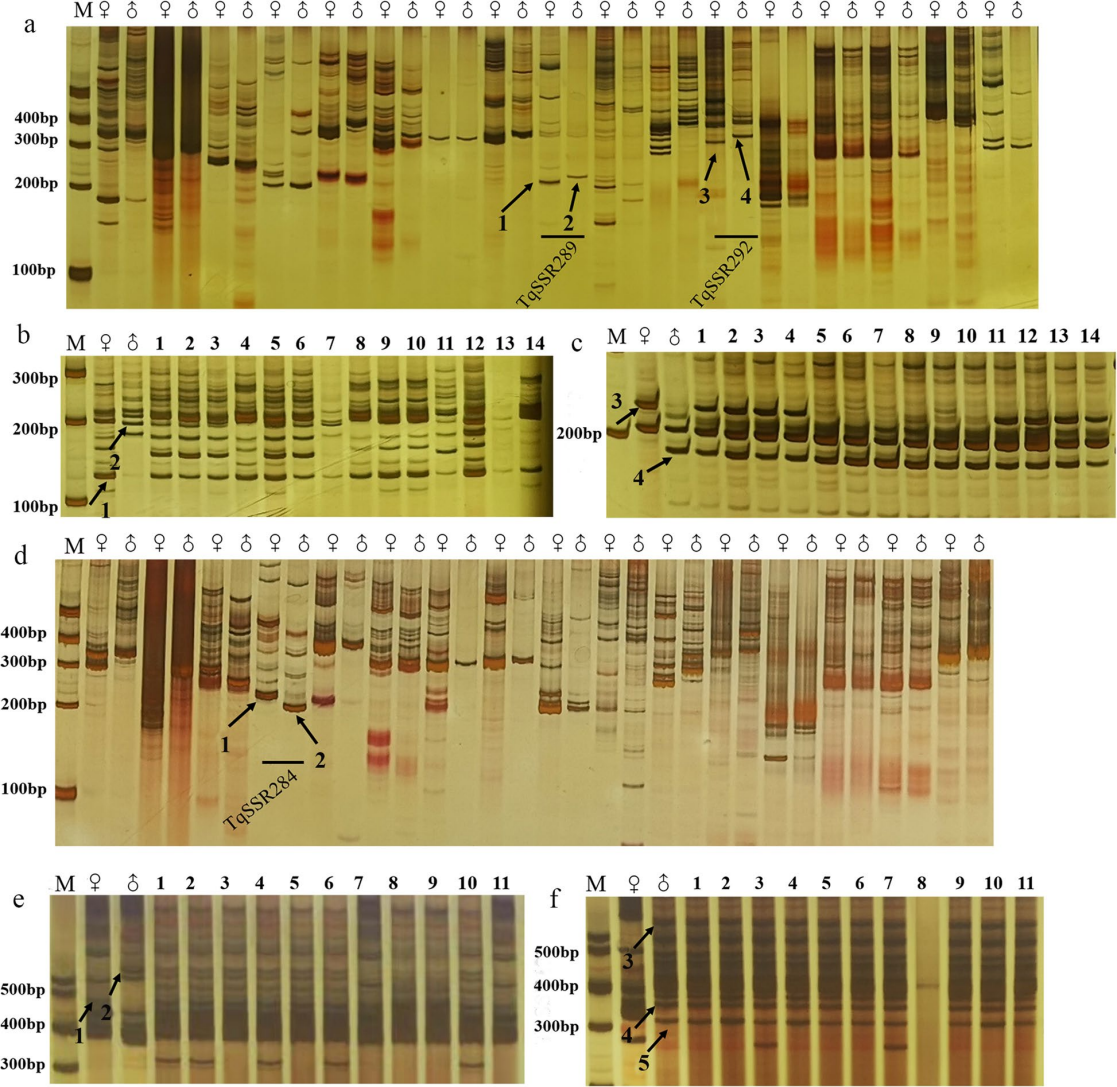
Genotyping of the parents and F_1_ lines of two hybrid populations using SSR markers. **a** Amplification patterns of parents of the Tl × Tvf cross. **b, c** Genotyping of Tl, Tvf, and F_1_ hybrids with TqSSR119 (**b**) and TqSSR124 (**c**) markers. **d** Amplification patterns of parents of the Tve × Tq cross. **e, f** Genotyping of Tve, Tq and F_1_ hybrids with TqSSR170 (**e**) and TqSSR104 (**f**) markers

Male-sterile (without pollen) and male-fertile (with pollen) thyme species were crossed as female and male parents, respectively, in different combinations. Finally, two F_1_ hybrid populations were obtained from two crosses: *T. longicaulis* × *T. vulgaris* ‘Fragrantissimus’ (Tl × Tvf, 14 lines) and *T. vulgaris* ‘Elsbeth’ × *T. quinquecostatus* (Tve × Tq, 11 lines) (Supplementary Table S1). To design SSR markers for the verification of F_1_ progenies, 300 primer pairs were screened by performing PCR amplification on the parental lines of the two crosses (Supplementary Table S2). Analysis of the PCR products revealed 1–2 polymorphic bands between the two parents of each cross. After many repetitions, the primers showing clear and stable banding patterns were selected (Fig. 3a, d).

Eighteen SSR markers were co-dominant in the Tl × Tvf population (Supplementary Table S3). For example, TqSSR289 amplified band 1 in the female parent T1 and band 2 in the male parent Tvf (Fig. 3a); TqSSR292 amplified band 3 in the female parent T1 and band 4 in the male parent Tvf (Fig. 3a). Similarly, 23 SSR markers were co-dominant in the Tve × Tq population (Supplementary Table S4). For example, TqSSR284 amplified band 1 in the female parent Tve and band 2 in the male parent Tq (Fig. 3d), so as to be used for the identification and verification of the hybrid progenies of the combination, accounting for 7.60% of 300 primer pairs. These co-dominant SSR primers were used to identify F_1_ individuals in the two hybrid populations. Progenies with complementary parental bands or only paternal-specific bands were true hybrids, and those with only maternal-specific bands were pseudo-hybrids or inbreds. Based on the genotyping results, 14 lines in the Tl × Tvf progeny (Fig. 3b, c), and 11 lines in the Tve × Tq progeny were identified as true hybrids (Fig. 3e, f).

### Determination of volatile organic compounds (VOCs) in the leaves of F_1_ hybrids and their parents

**Fig. 4:**
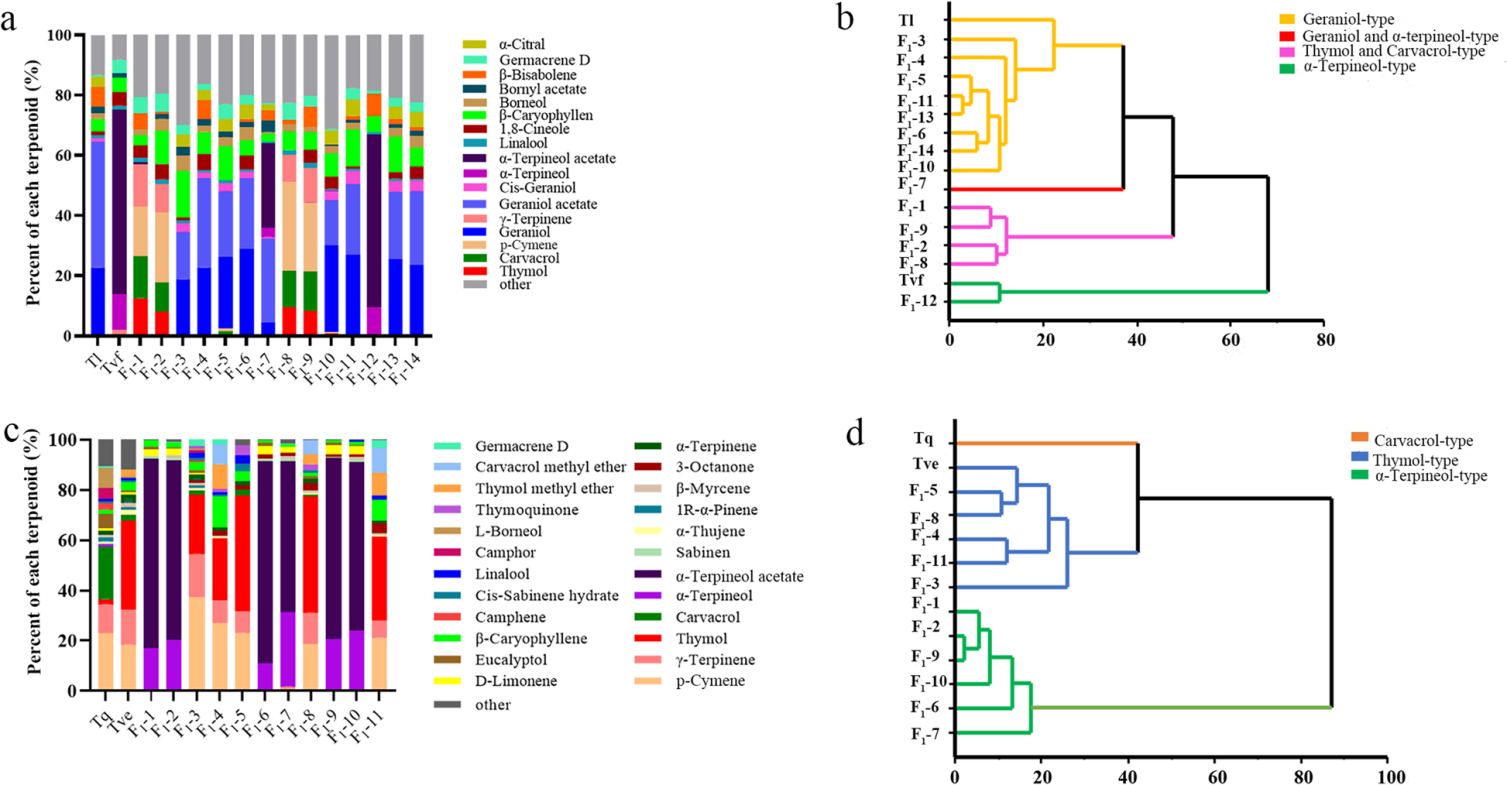
Analysis of volatile terpenoids in leaves of parents and F_1_ lines of two hybrid populations. **a** Histogram of volatile terpenoids in the leaves of Tl, Tvf, and their F_1_ progeny. **b** Cluster analysis of Tl, Tvf, and their F_1_ progeny, based on their terpenoid contents. **c** Histogram of volatile terpenoids in the leaves of Tve, Tq, and their F_1_ progeny. **d** Cluster analysis of Tve, Tq, and their F_1_ progeny, based on their terpenoid contents

Geraniol (22.75%) and geranyl acetate (41.75%) were the most abundant VOCs in the female parent Tl, and α-terpineol (11.76%) and α-terpineol acetate (61.37%) were the most abundant in the male parent Tvf (Fig. 4a; Supplementary Table S5). Among the 14 F_1_ lines derived from the Tl × Tvf cross, F_1_-1, F_1_-2, F_1_-8, and F_1_-9 showed the highest contents of thymol (12.51%, 8.03%, 9.55%, and 8.39%, respectively), carvacrol (13.93%, 9.72%, 12.17%, and 12.92%, respectively), *p*-cymene (16.52%, 23.22%, 29.39%, and 23.01%, respectively), and γ-terpinene (14.16%, 9.50%, 9.02%, and 11.36%, respectively). Additionally, lines F_1_-3, F_1_-4, F_1_-5, F_1_-6, F_1_-10, F_1_-11, F_1_-13, and F_1_-14 showed the highest contents of geraniol (18.17%, 22.75%, 23.79%, 28.98%, 28.84%, 26.91%, 25.44%, and 23.57%, respectively) and geranyl acetate (15.76%, 29.59%, 21.89%, 23.41%, 15.05%, 23.60%, 22.50%, and 24.56%, respectively). Thus, the essential oil compositions of these progenies were biased toward the female parent Tl. VOCs with high contents in line F_1_-7 were geraniol (4.48%), geranyl acetate (27.75%), α-terpineol (3.00%), and α-terpineol acetate (28.03%), thus representing a good aggregation of the dominant compounds found in the two parents. The most abundant compounds in line F_1_-12 were α-terpineol (9.07%) and α-terpineol acetate (57.44%), indicating a bias toward the male parent Tvf. Cluster analysis of the 17 main chemical compounds found in T1, Tvf, and their 14 F_1_ lines showed that lines F_1_-3, F_1_-4, F_1_-5, F_1_-6, F_1_-10, F_1_-11, F_1_-13, and F_1_-14, which clustered with the female parent Tl, were geraniol-type; F_1_-12, which grouped with the male parent Tvf, was α-terpineol-type; lines F_1_-1, F_1_-2, F_1_-8, and F_1_-9 were thymol and carvacrol polymerization-type; and line F_1_-7 was geraniol and α-terpineol polymerization-type (Fig. 4b).

Next, we analyzed the parents and progeny of the Tve × Tq combination (Fig. 4c; Supplementary Table S6). Thymol (35.43%), *p*-cymene (18.42%), and γ-terpinene (13.96%) were the most abundant in the female parents Tve, and carvacrol (20.74%), *p*-cymene (23.23%), and γ-terpinene (11.45%) were the most abundant in the male parent Tq. Among the 11 Tve × Tq F_1_ lines, F_1_-3, F_1_-4, F_1_-5, F_1_-8, and F_1_-11 showed the highest contents of thymol (23.57%, 24.71%, 46.19%, 46.38%, and 33.26%, respectively), *p*-cymene (37.42%, 27.03%, 23.16%, 18.56%, and 21.14%, respectively), and γ-terpinene (17.14%, 9.03%, 8.55%, 12.58%, and 6.85%, respectively). The compositions of these F_1_ lines were biased towards the female parent Tve, and the thymol contents of F_1_-5 and F_1_-8 were higher than that of the female parent, accounting for 46.19% (F_1_-5) and 46.38% (F_1_-8) of the progeny plants. Lines F_1_-1, F_1_-2, F_1_-6, F_1_-7, F_1_-9, and F_1_-10 showed the highest contents of α-terpineol (16.84%, 20.34%, 11.25%, 29.45%, 20.72%, and 23.98%, respectively) and α-terpineol acetate (75.72%, 71.58%, 80.44%, 60.21%, 72.17%, and 67.11%, respectively), and the sum of the proportions of each of these two compounds (α-terpineol and α-terpineol acetate) in the six above-mentioned F_1_ lines was more than 90%. Thus, α-terpineol and α-terpineol acetate were the absolute dominant compounds, although these compounds were not dominant in the parents; neither one of the two compounds was detected in the female parent Tve, and only α-terpineol was detected in minor amounts (0.95%) in the male parent Tq. Cluster analysis revealed that, among the 11 progeny lines, F_1_-3, F_1_-4, F_1_-5, F_1_-8, and F_1_-11 were thymol-type, while F_1_-1, F_1_-2, F_1_-6, F_1_-7, F_1_-9, and F_1_-10 were α-terpineol-type (Fig. 4d).

### Bioinformatics analysis and screening of TPSs in thyme

**Fig. 5:**
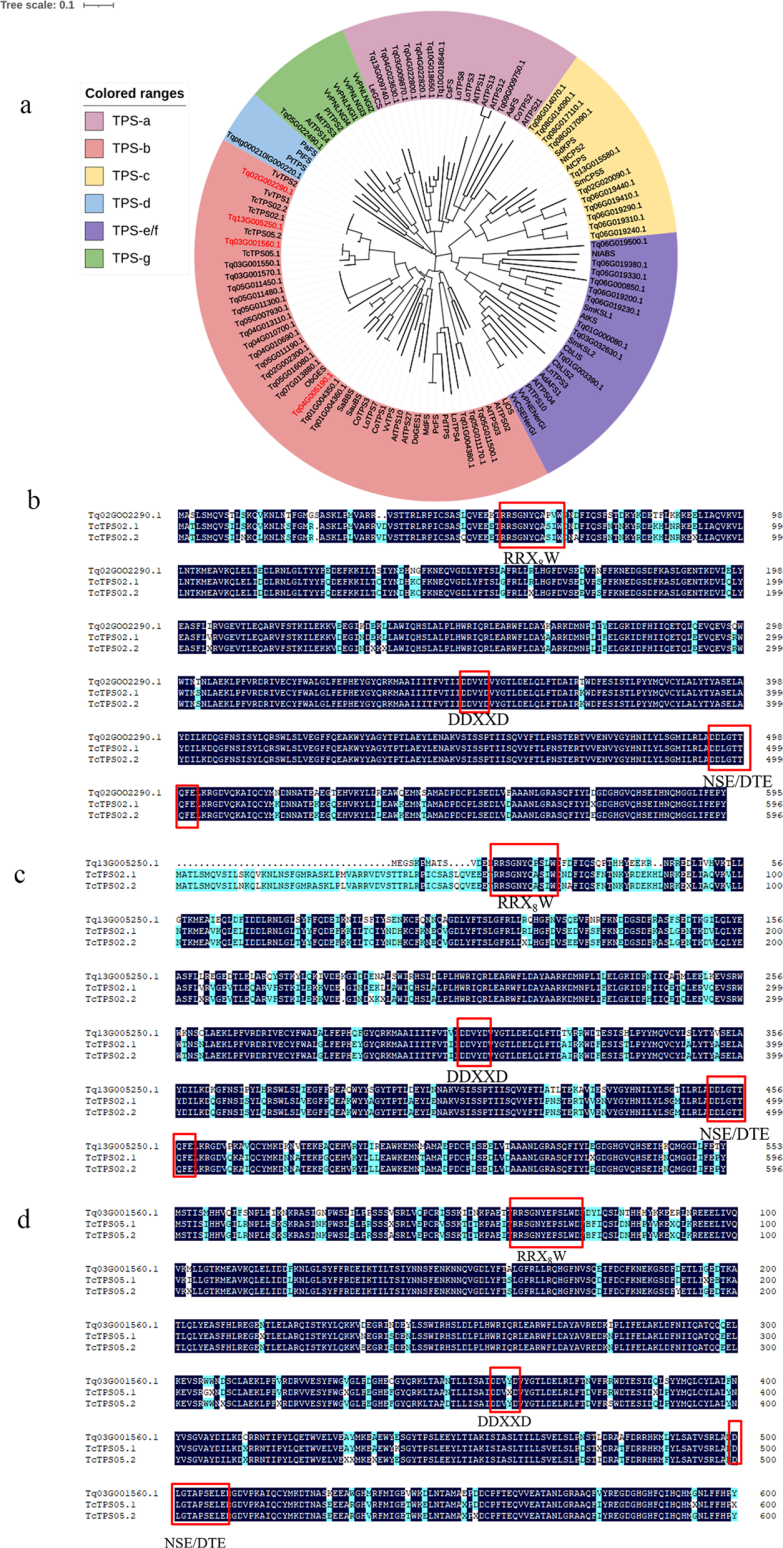
Phylogenetic analysis and amino acid sequence alignment of TPS proteins in thyme species. **a** Phylogenetic analysis of 52 TPSs in thyme. **b,c** Alignment of the deduced amino acid sequences of *Tq02G002290.1* (**b**) and *Tq13G005250.1* (**c**) with the reported amino acid sequences of γ-terpinene synthases (*TcTPS02.1* and *TcTPS02.2*; Lima et al., 2013). **d** Alignment of the deduced amino acid sequence of *Tq03G001560.1* with the reported amino acid sequences of α-terpineol synthases (*TcTPS05.1* and *TcTPS05.2*; Lima et al., 2013). Red boxes indicate the conserved motifs (RRX8W, DDXXD, and NSE/DTE) of TPSs

TPSs convert GPP and FPP into the basic skeleton of monoterpenes (C10) and sesquiterpenes (C15), respectively. We determined the volatile terpenes in the leaves of Tl × Tvf and Tve × Tq populations. The chemical types found in the Tl × Tvf population were geraniol-type, geraniol/α-terpineol-type, thymol/carvacrol-type, and α-terpineol-type, while those found in the Tve × Tq population were carvacrol-type, thymol-type, and α-terpineol-type (Fig. 4b, d). Considering that TPS is the key enzyme involved in plant terpenoid biosynthesis, we aimed to identify the main TPSs, such as γ-terpinene synthase, geraniol synthase, and α-terpineol synthase, that determine the essential oil composition of the two F_1_ progenies. Based on the results of *T. quinquecostatus* whole-genome sequencing [36], 69 *TPS* sequences were selected according to gene function annotation, of which 17 sequences were removed based on conserved domain analysis. A phylogenetic tree was constructed using the remaining 52 TPS sequences of *T. quinquecostatus* and the TPS sequences of other species (Fig. 5a; Supplementary Table S7). The results showed that 22 TPS sequences belonged to the TPS-b family, which contains monoterpene synthases. Among these sequences, *Tq02G002290.1* and *Tq13G005250.1* were predicted to encode γ-terpinene synthases; *Tq03G001560.1* encoded α-terpineol synthase; and *Tq04G005190.1* maybe encoded geraniol synthase. These genes were compared with their homologs in *T. caespititius* [29]; comparison of *Tq02G002290.1* and *Tq13G005250.1* with the reported γ-terpinene synthase genes *TcTPS02.1* and *TcTPS02.2* in *T. caespititius* revealed 96.60% and 89.35% sequence similarities, respectively (Fig. 5b, c), and *Tq03G001560.1* showed 95.51% sequence similarity with the α-terpineol synthase genes *TcTPS05.1* and *TcTPS05.2* in *T. caespititius* (Fig. 5d). *Tq04G005190.1* was compared with the *Ocimum basilicum* the geraniol synthase gene *ObGES* gene [40] (Supplementary Fig. S3), which revealed 68.90% sequence similarity. Alignment of the deduced amino acid sequences of *Tq02G002290.1*, *Tq13G005250.1*, and *Tq03G001560.1* revealed three characteristic TPS domains, including RRX8W, DDXXD, and NSE/DTE (Fig. 5b–d), while *Tq04G005190.1* showed two characteristic TPS domains, including DDXXD and NSE/DTE (Supplementary Fig. S3).

**Fig. 6:**
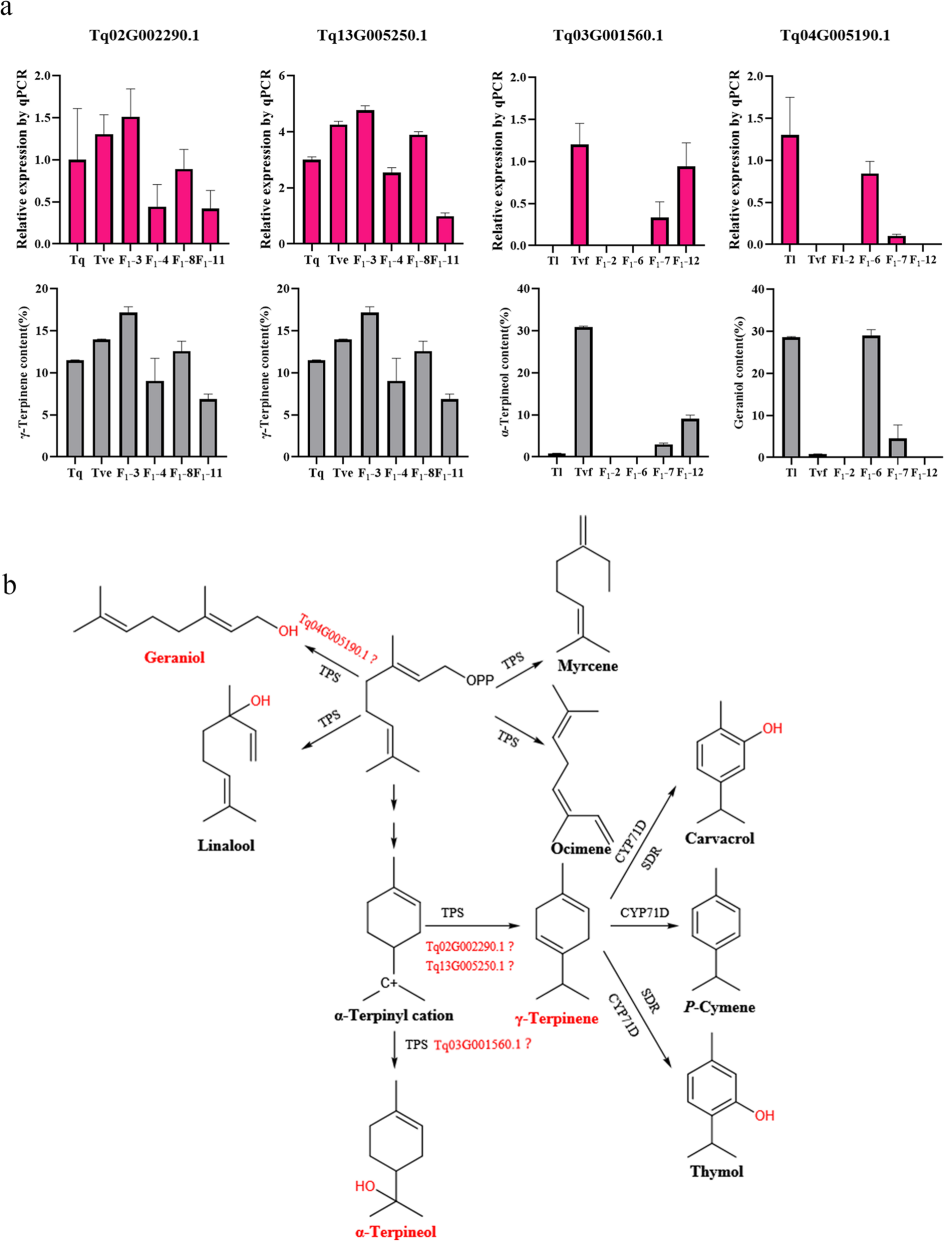
*TPS* gene expression analysis, and biosynthesis pathway of some monoterpenoids. **a** Expression of *TPS* genes in the parents and F_1_ progenies of two hybrid populations (Tve × Tq and Tl × Tvf). **b** Biosynthesis pathway of some monoterpenoids

The chemical types of the male parent (Tq) and female parent (Tve) of Tve × Tq were carvacrol- and thymol-type, respectively. Since γ-terpinene is the catalytic precursor of thymol and carvacrol, we verified the γ-terpinene synthase function of *Tq02G002290.1* and *Tq13G005250.1* by monitoring the expression levels of these genes in Tve, Tq, and their progenies F_1_-3 and F_1_-4 by qRT-PCR (Fig. 6a). In lines F_1_-3 and F_1_-4, the relative expression levels of *Tq02G002290.1* and *Tq13G005250.1* were consistent with the relative content of γ-terpinene, suggesting that *Tq02G002290.1* and *Tq13G005250.1* catalyze γ-terpinene biosynthesis (Fig. 6a). Similarly, to better verify the α-terpineol synthase and geraniol synthase functions of *Tq03G001560.1* and *Tq04G005190.1*, respectively, we examined the expression levels of these two genes in Tl (geraniol-type), Tvf (α-terpineol-type), and their progenies F_1_-6 and F_1_-12 by qRT-PCR (Fig. 6a). In lines F_1_-6 and F_1_-12, the expression levels of both genes were consistent with the relative contents of α-terpineol and geraniol, indicating that *Tq03G001560.1* and *Tq04G005190.1* catalyze α-terpineol and geraniol biosynthesis, respectively (Fig. 6a).

## Discussion

### Phenotypic evaluation of thyme species and construction of two F_1_ hybrid populations

In this study, the essential oil yield of erect-type thyme was higher than that of creeping-type thyme, and there was a positive correlation between the density of glandular trichomes and essential oil yield (Fig. 1e, f). Some wild Chinese thyme species showed a long flowering period of up to four months, while some European thyme species showed a short flowering period of one month (Table 1). The tillering capacity of creeping-type species was greater than that of erect-type species. In addition, the harvest time, extraction method, and stem/leaf ratio of thyme species likely affected their essential oil yield. In this study, the type and relative content of VOCs in the essential oil varied significantly among the 10 different thyme species, which further illustrated the diversity of the *Thymus* genus (Table 2). A previous study reported that the essential oil composition of a given thyme species and the contents of individual VOCs vary among different environments [40]. Additionally, extraction methods affect the composition and content of essential oil [17]. Therefore, the effects of species, region, and extraction method should be considered when studying the content and composition of thyme essential oil (Supplementary Fig. S1; Table 2). A certain correlation was also detected between the color and composition of thyme essential oil. Essential oil extracted from α-terpineol-type thyme was milky white whereas that extracted from geraniol-type thyme was light yellow. Essential oils extracted from thymol-type and carvacrol-type thyme species were golden yellow. The most well-studied volatile compounds of Chinese and European thyme species are thymol and carvacrol, since these two compounds are the two most abundant VOCs in most thyme species [4, 8, 9]. In this study, the chemical types of the seven European thyme species were mainly thymol-, geraniol-, and α-terpineol-type, while those of the three wild Chinese thyme species were carvacrol- and thymol-type.

The phenomenon of male sterility in plants enables cross breeding while avoiding complicated process of emasculation. Darwin reported that thyme plants in southern England produce two kinds of flowers: one with intact male and female organs, and the other (a small flower) with no or completely cut anthers; the latter are completely male-sterile [41]. In different ecological environments, the proportion of female plants in different thyme species is more than 50% [42]. Because thyme flowers are very small in size, emasculation and crossing are difficult. Therefore, if some thyme plants possess female reproductive organs, crossing is very easy. Among the 10 thyme species investigated in this study, Tr, Tve, Tg, Tt, Ts, and Tl were male-sterile, while Tq, Tqp, Tm, and Tvf were male-fertile. Therefore, Tve and T1 were used as female parents, and Tq and Tvf were used as male parents to generate two hybrid thyme populations. Male sterility is jointly determined by genetic and environmental factors. The male-sterile thyme lines used in this study were introduced from Europe; therefore, the phenomenon of male sterility in these lines may be caused by maladjustment to the new environment.

Chinese thyme plants are widely distributed in the northwest, north, and northeast regions in China [15]. Therefore, the research on Chinese thyme species should be intensified, and wild Chinese thyme species should be crossed with European thyme species to breed new species suitable for ornamental, medicinal, and edible applications to promote the development of the thyme planting industry. Hybrid breeding is an important means of germplasm resource innovation [12]. The advantages of both parents can be introgressed into a single genetic background through interspecific hybridization. In this study, new thyme germplasm resources were generated by crossing different chemical types and plant types of thyme.

### SSR marker development and application

Hybrid breeding is an important means of germplasm resource innovation. The advantages of parents can be combined in a single genetic background through interspecific hybridization. In this study, new thyme germplasm resources were generated by hybridizing different chemical types and plant types. SSR markers are simple, time-saving, cost-effective, reproducible, and stable, and therefore have been widely used for the identification of the hybrid progenies in different species [16, 43]. Early identification and selection of hybrid progeny is an important link in cross breeding. Therefore, molecular marker technology, combined with morphological observation, can be used to identify hybrid progenies with high accuracy.

The SSR-based genotyping process first involves the screening of co-dominant SSR primers in parents, followed by the identification of progenies [43]. However, there may be errors in SSR marker-based identification of hybrid progenies using a single primer pair. For example, in this study, SSR primers TqSSR119 and TqSSR124 were used to identify F_1_ progenies derived from the Tl × Tvf cross. Analysis of the same sample using different primer pairs can produce different results, possibly because of the high heterozygosity of thyme, lead to some of the parents can’t dominance or appear some non-parental bands. This may also be caused by the process of meiotic division during gamete formation; exchange of DNA between homologous chromosomes during recombination at marker loci can lead to the disappearance of bands in progenies.

Generally, the phenotype of F_1_ progenies is intermediate between that of the two parents or biased towards the phenotype of the female or male parent, which also provides the possibility of breeding excellent new varieties [43]. In this study, among the 14 Tl × Tvf F_1_ lines, eight lines showed the same VOC profiles in leaves as their female parent Tl; one line was similar to the male parent Tvf; and five lines were dissimilar to both parents. This conclusion was supported by the 11 F_1_ lines derived from the Tve × Tq cross; five of these F_1_ lines showed the same leaf VOC profiles as their female parent Tve, and the remaining six lines were different from both their parents. However, some F_1_ progenies also showed some volatile compounds that were superior to the both parents or not found in either parent; for example, thymol and carvacrol were found in the progeny of the Tl × Tvf cross, and α-terpineol and α-terpineol acetate were found in the Tve × Tq population. These results suggest that new varieties with related VOC profiles could be developed in thyme through cross breeding. Heterosis may also be present, and the greater the phenotypic differences between parents, the stronger is the heterosis expected to be in their progeny.

### Bioinformatics analysis and screening of TPSs in thyme

Monoterpenoid biosynthesis begins with GPP, which is the precursor of all monoterpenoids, and yields α-terpinyl cations, which are highly unstable intermediates that can then be converted to specific monoterpenoids by certain monoterpene synthases such as γ-terpinene and α-terpineol [44]. GPP also serves as the synthetic precursor of geraniol, linalool, myrcene, and ocimene, which are formed through catalysis by different monoterpene synthases. In addition, cytochrome P450 monooxygenase 71D (CYP71D) proteins subfamily and short-chain dehydrogenases/reductases (SDRs) are involved in further modification of the γ-terpinene framework to produce *p*-cymene, thymol, and carvacrol [36]. The synthetic pathway of some monoterpenes is shown in Fig. 6b. Functions of the γ-terpinene synthase gene *TcTPS02* and α-terpineol synthase gene *TcTPS05* were previously validated in *T. caespititius* [29]. Similarly, functions of the γ-terpinene synthase gene *TvTPS2* and three cytochrome P450 genes (*TvCYP71D179*, *TvCYP71D180*, and *TvCYP71D507*) were validated in *T. vulgaris* [45, 46].

The results of qRT-PCR analysis showed that the screened *TPSs* were expressed in hybrid thyme progenies, and these results were consistent with the relative contents of the TPS-catalyzed products in thyme. The expression levels of two γ-terpinene synthase genes *Tq02G002290.1* and *Tq13G005250.1* were verified in the progeny derived from the cross between Tve and Tq, whose chemotypes were thymol- and carvacrol-type, respectively. Similarly, the expression levels of α-terpineol synthase gene *Tq03G001560.1* and geraniol synthase gene *Tq04G005190.1* were validated in the progeny of Tl and Tvf, whose chemotypes were α-terpineol- and geraniol-type, respectively. Additionally, the relative expression levels of *Tq02G002290.1*, *Tq13G005250.1*, *Tq03G001560.1*, and *Tq04G005190.1* were consistent with the relative contents of catalytic products in some F_1_ lines. Therefore, this method enables only a preliminary screening of gene function and provides a basis for further gene function verification. The above results lay a strong foundation for the creation of new germplasm resources, construction of the genetic linkage maps, mapping of quantitative trait loci (QTLs), and MAS, and provide insight into the mechanism of monoterpenoids biosynthesis in thyme.

## Conclusion

Thyme is a multi-purpose plant with a wide range of applications in the pharmaceutical, food, and cosmetic industries, owing to its high essential oil content. To obtain thyme essential oil with different terpene compositions, we cross wild Chinese thyme species with European thyme species, and new germplasm resources have developed using the conventional hybridization approach. Two F_1_ populations were obtained, simultaneously, SSR primers were developed based on the whole-genome sequence of *T. quinquecostatus* to authenticate the F_1_ hybrids. Based on the chemotype of the parents and their F_1_ progenies, we examined the expression of genes encoding two γ-terpinene synthases, one α-terpineol synthase, and maybe one geraniol synthase in all genotypes by qRT-PCR. The results of this study provide a strong foundation for the creation of new germplasm resources, construction of the genetic linkage maps, and identification of QTLs responsible for the terpene compositions and essential oil. We screened monoterpene synthases in thyme to gain insight into the mechanism of monoterpenoids biosynthesis in thyme.

## Methods

### Plant materials

Three wild Chinese thyme species, including *T. quinquecostatus*, *T. quinquecostatus* var. *przewalskii*, *T. mongolicus*, and seven European thyme species, including *T. vulgaris* ‘Fragrantissimus’, *T. vulgaris* ‘Elsbeth’, *T. guberlinesis*, *T. serpyllum* ‘Aureus’, *T. thracicus*, *T. longicaulis*, and *T. rotundifolius*, were used in this study. *T. quinquecostatus* plants were collected from the Jinhekou village in the Hebei province of China in 2018. *T. quinquecostatus* var. *przewalskii* was collected in 2019 from the Small Wutai Jinhekou Scenic Spot in Yu County, Hebei, China. *T. mongolicus* was collected in 2018 from Chicheng County, Hebei, China. Three specimens (voucher nos. 2,582,781, 2,582,673, and 2,582,686) were obtained from the Chinese National Herbarium, Institute of Botany, Chinese Academy of Sciences. *T. vulgaris* ‘Fragrantissimus’, *T. vulgaris* ‘Elsbeth’, *T. thracicus*, and *T. longicaulis* were introduced from the Czech Republic (introduction no. 708–2015, 626–2015, 628–2015, and 709–2015, respectively). *T. guberlinensis* and *T. serpyllum* ‘Aureus’ were introduced from Hungary (introduction no. 002-2016 and 003-2016). *T. rotundifolius* was introduced from Germany (introduction no. 797–2016). Plants were grown on an experimental farm at the Institute of Botany, Chinese Academy of Sciences (IB-CAS), Beijing, China.

### Hybrid breeding design

Male-sterile (without pollen) thyme species were crossed as the female parent with male-fertile (with pollen) thyme species, with the essential oil composition and yield as the main breeding goals. Different cross combinations were designed, and the F_1_ hybrid combinations of thyme were constructed by cross breeding in 2020. Finally, two F_1_ populations derived from *T. longicaulis* (Tl) × *T. vulgaris* ‘Fragrantissimus’ (Tvf) and *T. vulgaris* ‘Elsbeth’ (Tve) × *T. quinquecostatus* (Tq) crosses were selected for further analysis.

### DNA extraction

Leaves were collected from the parents and F_1_ progenies of Tl × Tvf and Tve × Tq crosses, immediately frozen in liquid nitrogen, and stored at -80 °C. DNA was extracted from the frozen leaf samples using the DNA Secure Plant Kit (Tiangen, China). DNA concentration and quality were assessed by 1% agarose gel electrophoresis and with a 2.0 Fluorometer (Life Technologies, CA, USA).

### SSR genotyping

PCR was performed on the PCR system (Bio-Rad, Hercules, CA, USA) in a 10 µl reaction volume containing 2 µl (20 ng/µl) of genomic DNA, 3 µl of 2× Taq PCR Master Mix II (Tiangen, China), 2 µl of forward and reverse primer mixture, and 3 µl of ddH_2_O. The thermocycling conditions were as follows: 94 °C for 3 min; 6 cycles at 94 °C for 45 s, 55–65 °C for 1 min, and 72 °C for 1 min; 9 cycles of 94 °C for 45 s, 50–58 °C for 1 min, and 72 °C for 1 min; 19 cycles at 94 °C for 30 s, 50 °C for 30 s, and 72 °C for 1 min; and final extension at 72 °C for 5 min. PCR products were analyzed by electrophoresis on 8.0% (w/v) denaturing polyacrylamide gel in TBE buffer for 1 h on the DYY-6 C electrophoresis apparatus (Beijing Liuyi Instrument Factory, China) at a constant voltage of 220 V. DNA fragments were visualized by silver staining (Silver sequence staining reagents, Promega, Madison, USA) and sized with a 50 bp DNA ladder marker (Tiangen, China) [47]. SSR primer sequences are listed in Supplementary Table S2.

### RNA extraction and cDNA synthesis

Leaves of *T. longicaulis*, *T. vulgaris* ‘Fragrantissimus’, *T. vulgaris* ‘Elsbeth’, *T. quinquecostatus*, and their F_1_ lines were frozen in liquid nitrogen and stored at -80 ℃. Total RNA was extracted from the frozen leaves using the Easy Spin RNA extraction kit (Sangon Biotech, Shanghai, China). The isolated total RNA was treated with DNase I, and then purified with the RNA clean kit (Promega, Madison, WI, USA). The concentration of each RNA sample was determined using NanoDrop spectrophotometer (Thermo Fisher Scientific Inc., USA) and 2.0 Fluorometer (Life Technologies, CA, USA). RNA integrity was analyzed using Bioanalyzer 2100 (Agilent Technologies, Santa Clara, CA). Then, cDNA was synthesized using the HiScript Reverse Transcriptase Kit (Vazyme, China), according to the manufacturer’s instructions [33].

### Gene expression analysis by qRT-PCR

To analyze the expression patterns of γ-terpinene, α-terpineol, and geraniol synthase genes, the total RNA of *T. vulgaris* ‘Elsbeth’, *T. quinquecostatus*, *T. longicaulis*, *T. vulgaris* ‘Fragrantissimus’, and their F_1_ lines was isolated and reverse transcribed, as described above. To amplify four *TPS* genes (encoding two γ-terpinene synthases, one α-terpineol synthase, and one geraniol synthase), primers were designed using Primer3 (http://primer3.ut.ee) (Supplementary Table S8). Then, qRT-PCR was carried out on the CFX96 instrument (Bio-Rad, USA) with Ssofast EvaGreen Supermix Kit in a 20 µl reaction volume containing 0.8 µl of each primer, 1 µl of template cDNA, 10 µl of 2× T5 Fast qRT-PCR Mix (TSINGKE, China), 7 µl of ddH_2_O, and 0.4 µl of 50× ROX Reference Dye II (TaKaRa, China). The qRT-PCR protocol included 40 cycles of 95 ℃ for 30 s, 95 ℃ for 10 s, and 58 ℃ for 30 s, along with melting curve analysis. Primer sequence, amplicon length, amplification efficiency (%), and linear correlation coefficient (R^2^) corresponding to two internal reference genes (*18 S rRNA* and *β-actin*) are shown in Supplementary Table S8. Finally, the relative quantification of four *TPS* transcripts was performed using the internal reference gene *18 S rRNA.* Relative expression levels were calculated using the 2^−ΔΔCt^ method after transcript data normalization. All analyses were performed in triplicate [40].

### Essential oil extraction

The essential oil of 10 thyme species was isolated by steam distillation at 180–200 ℃ for 90 min. Essential oil yield (%) was calculated as volume (ml) of the isolated oil per 100 g of dry plant material. The isolated essential oil was dried using anhydrous sodium sulfate, and stored at 4 ℃ until needed for further analysis [11].

### Analysis of essential oil composition

The essential oil composition of 10 thyme species was analyzed by gas chromatography-mass spectrometry (GC-MS) using Agilent 7890 A-7000B gas chromatograph (Agilent, USA), equipped with Agilent 5975 C MS detector (Agilent, USA). Using the HP-5MS (30 m, 250 m ID, 0.25 μm film thickness) capillary column, volatiles were separated using the following temperature program: 5 min at 60 °C; increased to 220 °C at the rate of 4 °C/min; increased to 250 °C at the rate of 60 °C/min; hold at 250 °C for 5 min. The following parameters were used: injector and detector temperature, 250 °C; carrier gas, He; flow rate, 1 m/min; split ratio, 1:10; acquisition range, 50–500 m/z in electron-impact mode; ionization voltage, 70 eV; and injected sample volume, 1 µl. The determination of the content of each compound (%) was based on the normalization of GC peak areas. The identification of essential oil components was based on the comparison of retention indices (RIs), relative to a homologous series of n-alkanes (C7–C40), and mass spectra (MS) from the NIST (v14.0) library and data from scientific literature [48]. RIs were based on the equation:

$$\mathrm{RI}\:=\:100\mathrm Z\:+\:100\lbrack\mathrm{RT}(\mathrm x)\;-\;\mathrm{RT}(\mathrm z)\rbrack\;/\;\lbrack\mathrm{RT}(\mathrm z\:+\:1)\;-\;\mathrm{RT}(\mathrm z)\rbrack$$

where RT(x), RT(z), and RT(z + 1) for the composition, and the number of carbons Z and Z + 1 for the retention time of the normal alkane.

### Analysis of leaf VOC profiles of thyme

The leaf VOC profiles of *T. vulgaris* ‘Elsbeth’, *T. quinquecostatus*, *T. longicaulis*, *T. vulgaris* ‘Fragrantissimus’, and their F_1_ lines were detected via headspace solid-phase microextraction (HS-SPME). Briefly, 0.25 g of fresh leaf powder was weighed and immediately placed into a 20 ml headspace vial (Aligent, Palo Alto, CA, USA) containing 20 µl of internal standard solution (1 mg/ml, 3-Octanol, Cas#589-98-o, Aladdin, Shanghai, China). The vials were sealed using crimp-top caps with TFE-silicone headspace septa (Agilent, Palo Alto, CA, USA). Subsequently, each vial was immediately incubated at 40℃ for 30 min. Then, to absorb the volatiles, the headspace of each vial was exposed to 100 μm coating fiber polydimethylsiloxane (Supelco, Inc., Bellefonte, PA, USA) for 30 min. All VOCs on the coating fiber were analyzed by GC-MS using Model 7890 A GC instrument and 7000B mass spectrometer (Agilent, Palo Alto, CA, USA) [49].

The GC-MS conditions were as follows: injector temperature, 250 ℃; transfer line temperature, 250 ℃, respectively; column temperature, initially maintained at 50 ℃ for 3 min, gradually increased to 150 ℃ at 4 ℃/min for 2 min, and finally raised to 250 ℃ at 8 ℃/min for 5 min; carrier gas (helium) flow rate, 1 ml/min; injection, splitless mode; ionization voltage, 70 eV; source temperature, 250 ℃; and MS range, 35–500 m/z. Agilent MassHunter 5.0 was used to analyze the chromatograms and MS. VOCs were identified by comparing the retention times of individual peaks with those of authentic standards, and MS were determined based on the NIST v14.0 MS database and the data reported previously [50]. RIs were calculated using the following equation:

$$\mathrm{RI}\:=\:100\mathrm Z\:+\:100\lbrack\mathrm{RT}(\mathrm x)\;-\;\mathrm{RT}(\mathrm z)\rbrack\;/\;\lbrack\mathrm{RT}(\mathrm z\:+\:1)\;-\;\mathrm{RT}(\mathrm z)\rbrack$$

where RT(x), RT(z) and RT(z + 1) for the composition, and the number of carbons Z and Z + 1 for the retention time of the normal alkane.

### Density of glandular trichomes

Glandular trichomes were visualized using a stereomicroscope (Leica DVM6, Germany). The number of glandular trichomes within a certain leaf area was counted using the ImageJ software. The average glandular trichome density was calculated based on three plants.

### Statistical analysis

Data were expressed as the mean ± standard deviation of three biological replicates. Statistical analysis, including variance analysis, hierarchical clustering analysis, and correlation analysis, was performed using IBM SPSS Statistics for Windows, version 19.0 (Armonk, USA). Significant differences among the different genotypes were tested using a one-way analysis of variance (ANOVA), followed by Duncan’s multiple range test at 5% probability level (*p* ≤ 0.05).

## Supplementary Information

**Additional file 1:** **Supplementary Fig. S1.** Images of the essential oils of 10 different thyme species.

**Additional file 2:** **Supplementary Fig. S2.** Detection of simple sequence repeat (SSR) loci in the *Thymus quinquecostatus* genome.

**Additional file 3:** **Supplementary Table S1.** Phenotypic characteristics of the parents of two hybrid thyme populations.

**Additional file 4:** **Supplementary Table S2.** Primers used for the development of SSR markers.

**Additional file 5: Supplementary Table S3.** SSR primers used for the identification of F_1_ hybrids in the Tl × Tvf population.

**Additional file 6: Supplementary Table S4.** SSR primers used for the identification of F_1_ hybrids in the Tve × Tq population.

**Additional file 7: Supplementary Table S5.** Relative contents of volatile organic compounds (VOCs) in the leaves of Tl, Tvf, and their F_1_ progeny.

**Additional file 8: Supplementary Table S6.** Relative contents of VOCs in the leaves of Tve, Tq, and their F_1_ progeny.

**Additional file 9: Supplementary Table S7.** Reported amino acid sequences used to construct the TPS phylogenetic tree.

**Additional file 10: Supplementary Fig. S3.** Alignment of the deduced amino acid sequence of *Tq04G005190.1* with the reported amino acid sequence of geraniol synthase ObGES.

**Additional file 11: Supplementary Table S8.** Primer sequence, amplification length, amplification efficiency (%), and linear correlation coefficient (R^2^) of four TPS genes and two reference genes (*18 S rRNA and β-actin*).

**Additional file 12: Fig. 3-6** gels and blots.

## Data Availability

The raw sequence data and gene sequence information of *T. quinquecostatus* were deposited in NCBI under the project accession number PRJNA690675.

## Abbreviations

DMAPP: Dimethylallyl diphosphate
EO: Essential oil
FPP: Farnesyl diphosphate
GC: Gas chromatography
GC-MS: Gas chromatography-mass spectrometry
GPP: Geranyl diphosphate
Hi-C: Chromatin conformation capture
HiFi: High-fidelity
HS-SPME: Headspace solid-phase microextraction
IB-CAS: Institute of Botany, Chinese Academy of Sciences
IPP: Isopentenyl diphosphate
MAS: Marker-assisted selection
Mb: Megabases
MEP: 2-C-methyl-D-erythritol-4-phosphate
MVA: Mevalonate
PCA: Principal component analysis
PCR: Polymerase chain reaction
qRT-PCR: Quantitative real-time PCR
QTL: Quantitative trait locus
RIs: Retention indices
SSR: Simple sequence repeat
Tg: *T. guberlinesis* Iijin
Tl: *T. longicaulis*
Tm: *T. mongolicus*
TPS: Terpene synthase
Tq: *T. quinquecostatus*
Tqp: *T. quinquecostatus* var. *przewalskii*
Tr: *T. rotundifolius*
Ts: *T. serpyllum* ‘Aureus’
Tt: *T. thracicus*
Tve: *T. vulgaris* ‘Elsbeth’
Tvf: *T. vulgaris* ‘Fragrantissimus’
VOC: Volatile organic compound
