# Supplementary material for: Creation of new germplasm resources, development of SSR markers, and screening of monoterpene synthases in thyme
Source: BMC Plant Biol. 2023 Jan 6;23:13. doi: 10.1186/s12870-022-04029-2 (PMC9817278; doi:10.1186/s12870-022-04029-2)
Supplement: Supplementary file 1 — Additional file 1: Supplementary Fig. S1. Images of the essential oils of 10 different thyme species. [file 12870_2022_4029_MOESM1_ESM.docx]

**
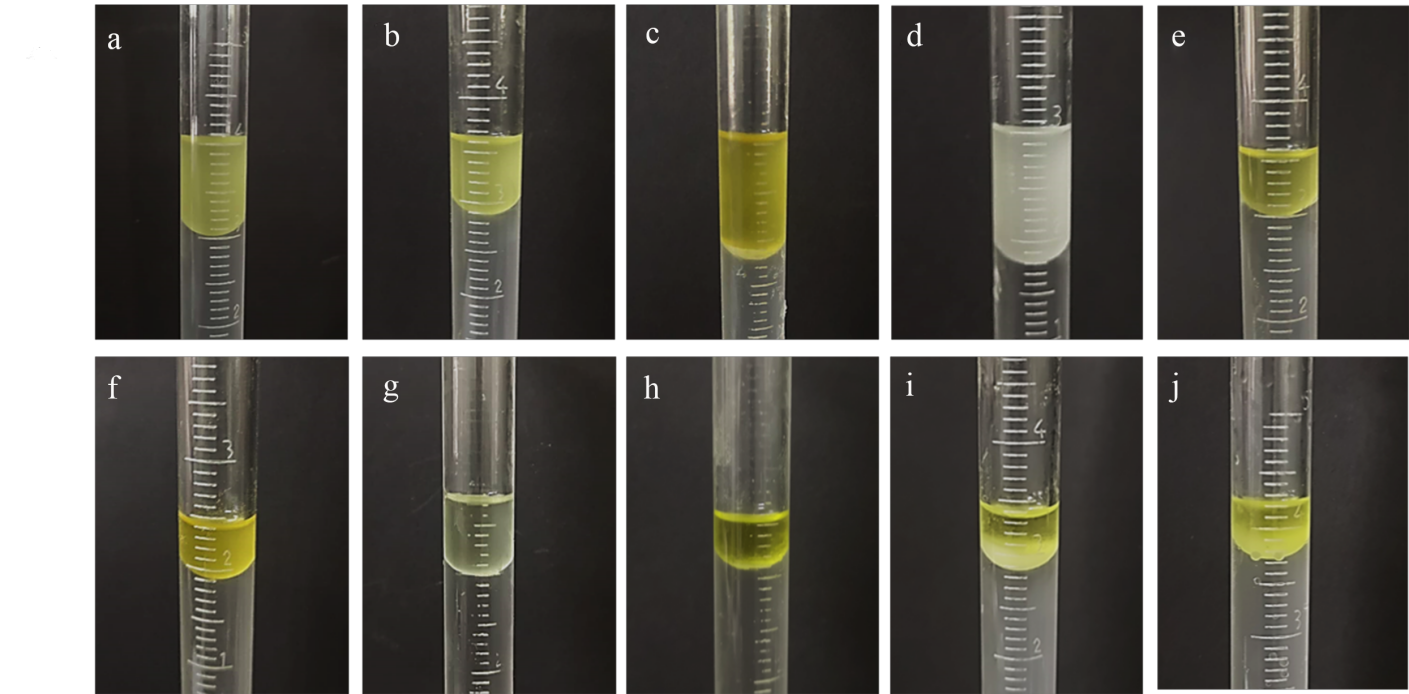
**

**Supplementary Fig. S1 Images of the essential oils of 10 different thyme species. a** Tr, *T.* *rotundifolius*; **b** Tve, *T.* *vulgaris* ‘Elsbeth’; **c** Tt, *T.* *thracicus*; **d** Tvf, *T. vulgaris* ‘Fragrantissimus’; **e** Ts, *T. serpyllum* ‘Aureus’; **f** Tg, *T. guberlinesis*; **g** Tl, *T. longicaulis*; **h** Tq, *T. quinquecostatus*; **i** Tqp, *T. quinquecostatus* var. *przewalskii*; **j** Tm, *T. mongolicus.*
