# Supplementary material for: Creation of new germplasm resources, development of SSR markers, and screening of monoterpene synthases in thyme
Source: BMC Plant Biol. 2023 Jan 6;23:13. doi: 10.1186/s12870-022-04029-2 (PMC9817278; doi:10.1186/s12870-022-04029-2)
Supplement: Supplementary file 2 — Additional file 2: Supplementary Fig. S2. Detection of simple sequence repeat (SSR) loci in the Thymus quinquecostatus genome. [file 12870_2022_4029_MOESM2_ESM.docx]

**
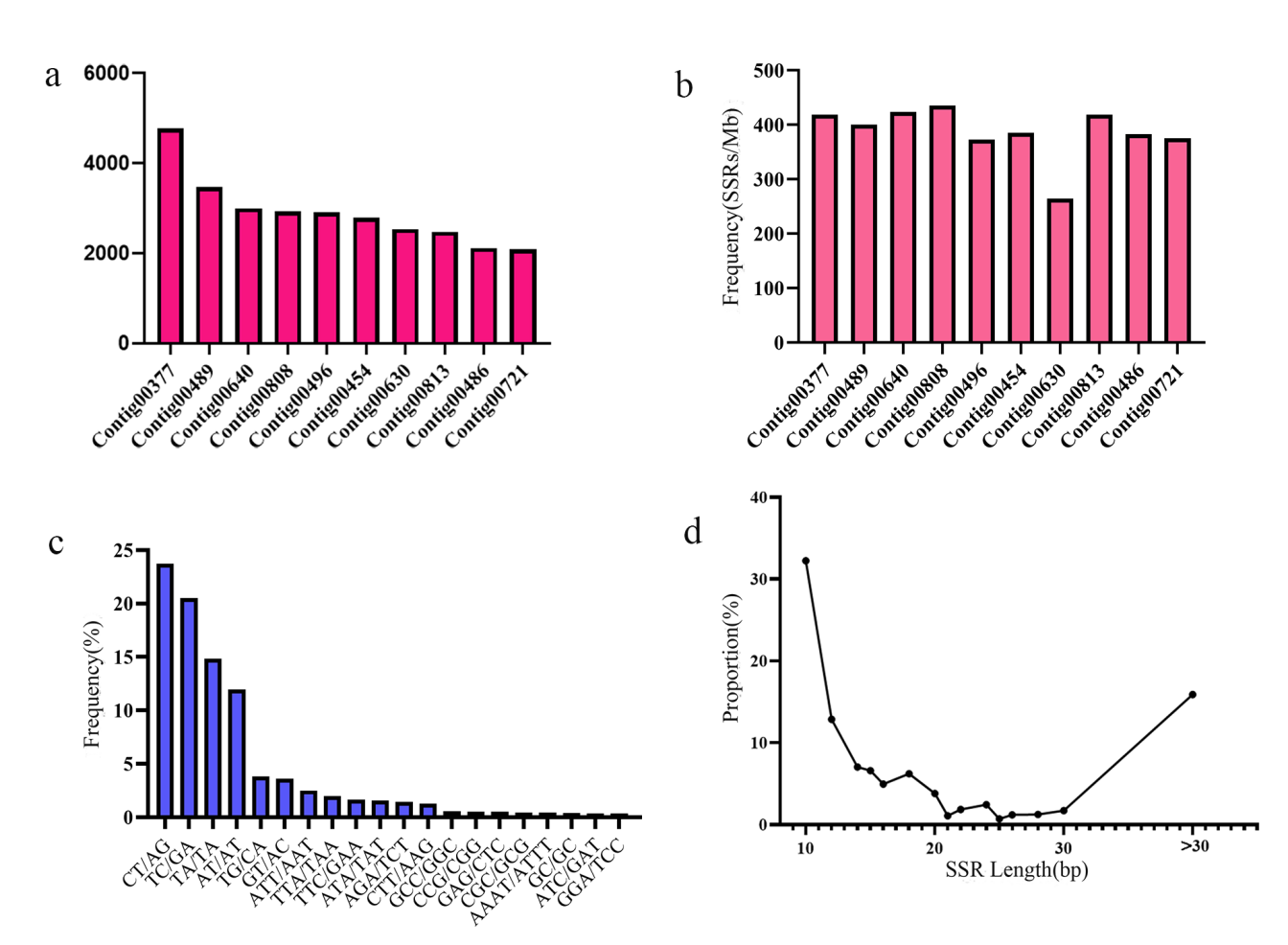
**

**Supplementary Fig. S2 Detection of simple sequence repeat (SSR) loci in the *Thymus quinquecostatus* genome. a** Number of SSR loci in the top 10 longest contigs. **b** Frequency of SSR loci in the top 10 longest contigs. **c** Type and frequency of di-, tri-, and tetranucleotide repeats at the SSR loci in the *T. quinquecostatus* genome. **d** SSR length distribution in the *T. quinquecostatus* genome.
