# Supplementary material for: Creation of new germplasm resources, development of SSR markers, and screening of monoterpene synthases in thyme
Source: BMC Plant Biol. 2023 Jan 6;23:13. doi: 10.1186/s12870-022-04029-2 (PMC9817278; doi:10.1186/s12870-022-04029-2)
Supplement: Supplementary file 3 — Additional file 3: Supplementary Table S1. Phenotypic characteristics of the parents of two hybrid thyme populations. [file 12870_2022_4029_MOESM3_ESM.docx]

**Supplementary Table S1 Phenotypic characteristics of the parents of two hybrid thyme populations.**

| **Phenotype** | **Population 1^a^** | | **Population 2^b^** | |
| --- | --- | --- | --- | --- |
|  | **♀ Tl** | **♂ Tvf** | **♀ Tve** | **♂ Tq** |
| **Chemotype** | geraniol | α-terpineol | carvacrol | thymol |
| **Plant type** | creeping | erect | erect | creeping |
| **Sterility** | male-sterile | male-fertile | male-sterile | male-fertile |
| **Oil yield** | 0.70% | 1.25% | 0.70% | 0.40% |
| **No. of F_1_ lines** | 14 | | 11 | |

^a^ Tl, *T. longicaulis*; Tvf, *T. vulgaris* ‘Fragrantissimus’.

^b^ Tve, *T.* *vulgaris* ‘Elsbeth’; Tq, *T. quinquecostatus.*
