# Supplementary material for: Creation of new germplasm resources, development of SSR markers, and screening of monoterpene synthases in thyme
Source: BMC Plant Biol. 2023 Jan 6;23:13. doi: 10.1186/s12870-022-04029-2 (PMC9817278; doi:10.1186/s12870-022-04029-2)
Supplement: Supplementary file 4 — Additional file 4: Supplementary Table S2. Primers used for the development of SSR markers. [file 12870_2022_4029_MOESM4_ESM.docx]

**Supplementary Table S2 Primers used for the development of SSR markers.**

| **Primer** | **Forward primer sequence** | **Tm (℃)** | **Reverse primer sequence** | **Tm (℃)** |
| --- | --- | --- | --- | --- |
| **TqSSR001** | CTTCTCCTCCCCGTCAATCT | 60.586 | GAGGGAAGGGAGAAGAATCG | 60.147 |
| **TqSSR002** | TCCGACCCACAGATCTGATTA | 60.467 | CAGCCCAAATCCATTACGTC | 60.331 |
| **TqSSR003** | AGTCACGCAATGCATCAAAA | 60.265 | TCCTCCACTCTGCAACAAAA | 59.415 |
| **TqSSR004** | TGATTGGGGTTGTGAAGACA | 59.935 | TGTTGTGTGTTTTGCTGCTG | 59.504 |
| **TqSSR005** | TATGGGTCCTCTCCGACAAG | 60.065 | ACTTAAGCCTGAGCCCCAAT | 60.096 |
| **TqSSR006** | TCCCTTTATAAGTGGGCTGAA | 58.705 | CTAGCGGGGATTTCATGTTT | 59.043 |
| **TqSSR007** | AGCACAAATTGAACGGGGTA | 60.365 | TGTGGCCGACCATAATTTAAC | 59.712 |
| **TqSSR008** | CGCGTGCATTAAATCCAAA | 60.598 | CGGTGTAGATTAACTGGTGCAT | 59.044 |
| **TqSSR009** | AAGATCCTCCCCATCTCCAG | 60.412 | TTCAACTCACCCACTTGCAG | 59.873 |
| **TqSSR010** | GTAGGTAGTGGCTCGCTTCG | 60.037 | CGCTTGAGTGTGTTTTCCAA | 59.881 |
| **TqSSR011** | CTCAGGCTTGGAACCGTAAC | 59.734 | GGTGTTCCTCATGCAACTCTT | 59.201 |
| **TqSSR012** | CGAGGTTGATGACAAACGTG | 60.152 | CACGCGTTCTTTGTCGTTTA | 59.911 |
| **TqSSR013** | GAGGGAATACGCAGAACGAA | 60.214 | CCCATTTTACCCTCCATCCT | 60.011 |
| **TqSSR014** | TGGAGAAATTCCAGCCAAAA | 60.555 | TGGCTGAAACAAGACTCGAA | 59.566 |
| **TqSSR015** | ACCGTCCACCGTATCTAAGC | 59.080 | TTGTTTCCTACCCTACCGACA | 59.480 |
| **TqSSR016** | ACTCCCTGCAACCTTCTCCT | 60.252 | GTTCGGGATGAAAAGTCTCG | 59.670 |
| **TqSSR017** | CAGTGTTTCAACCGTTGTCG | 60.190 | GATCAGTCAATCCAAGAGTAATCC | 58.163 |
| **TqSSR018** | GGTGTTCCTCATGCAACTATTC | 58.580 | CCTAAGGCTTGGAACCGTAA | 59.221 |
| **TqSSR019** | AAATCCGCCAGCATGTTATC | 59.929 | CGCCATGAATTCTTGTTGAA | 59.664 |
| **TqSSR020** | GCTTTAGGTAGCCAAGAACACA | 58.588 | TGTTGAGCTGATGGAATTCTG | 58.862 |
| **TqSSR021** | TCGTACTCGGGAAGAAGCAT | 59.836 | CTTCGCTGCTGGAGCTATTT | 59.752 |
| **TqSSR022** | GCATGAGAGGAGTGTTGGTG | 59.261 | GTGAAATGGGACGATCCAAA | 60.702 |
| **TqSSR023** | TCTCTCCACCGACCCAATAC | 59.927 | CGGAGAAGAAAAGGCAACAG | 59.986 |
| **TqSSR024** | TGTGCCTCGTCAAAATTCAA | 60.234 | GCTTCCTATGCTCTCCGTTC | 59.034 |
| **TqSSR025** | GAGGAATGAGGAGGGAGGAG | 60.149 | GTGGGGTTCAATTGGTCAAC | 60.073 |

**Continued Supplementary Table S2**

| **Primer** | **Forward primer sequence** | **Tm (℃)** | **Reverse primer sequence** | **Tm (℃)** |
| --- | --- | --- | --- | --- |
| **TqSSR026** | ACCCAATCTTTGTCGCATTC | 59.939 | TCGATACCACCCGATTCATT | 60.155 |
| **TqSSR027** | CTCAGTTGGGCCCAGTATGT | 59.989 | TTCTGACGTTGGATGGACAA | 60.088 |
| **TqSSR028** | ATTCAGGCGTGGGATATCAA | 60.296 | CCATACCATGTGTTTGGCATA | 59.172 |
| **TqSSR029** | CGGATCCGCAGTGCTATTAC | 60.626 | TGTCGGTTGTTTCTGGTTGA | 60.128 |
| **TqSSR030** | GGGCGATGCATAGGAAGATA | 60.023 | AAACTCCATGCAGCCAAGTC | 60.263 |
| **TqSSR031** | TCGGAGAAAGATGAGGGAGA | 59.879 | TGGACGAAAATACCCCTGAA | 60.301 |
| **TqSSR032** | GGGAACCCGATTATGTCAGA | 59.750 | ACAGAGGAAGGAGCAAAGCA | 60.134 |
| **TqSSR033** | CCTTGGCGTACTGTTCCATT | 59.993 | CACGGAGCTTTGCACTCATA | 60.011 |
| **TqSSR034** | GCATCAAAGAGAACGCAATC | 58.451 | CCGTGGAAATCCTTCCTCTA | 59.122 |
| **TqSSR035** | CTGCTTCTTTGCTGATGCTG | 59.888 | GGAAAATGAAGGGGAGAAGG | 59.875 |
| **TqSSR036** | CCCCTTTACAAGTGAATCCTTA | 57.332 | TTCGCAATTTGGGTGATGTA | 59.930 |
| **TqSSR037** | TTCTTGCTTGAGTGCCTCCT | 60.134 | CCTTTCACCATCCTCTTCCA | 60.042 |
| **TqSSR038** | ACGGCGCGATATATTTTCTG | 60.082 | ACCGGCTCTCATGATTTCTG | 60.218 |
| **TqSSR039** | CGGATGAAAGGAGGTAAATGA | 59.030 | GCATCTTTCGCATTTTAGCA | 59.056 |
| **TqSSR040** | AGAGGAGGAGGAGGAGAACG | 59.945 | CTCTTCGTTGGCGAAGAAAC | 59.993 |
| **TqSSR041** | TGTAATGCCCACTCCGTGTA | 59.988 | ACGCCCAATTCACAAAATTC | 59.807 |
| **TqSSR042** | ATTTCTTTGCCGTTGAGCAT | 59.713 | CTGGACCGGATCTACCAAAA | 59.926 |
| **TqSSR043** | GACTTCTCTATCTTGCCGCTCT | 59.286 | AAAAGAAAGCGCTAGGAGGA | 58.353 |
| **TqSSR044** | TTTGCCTTGTTTTGGGTTTC | 59.953 | GAGGAAATGGTGGGATTTCA | 59.727 |
| **TqSSR045** | CCGTCGATCAAGTACAGCAA | 59.864 | TTTAATCCTTGCACCCGTTG | 60.856 |
| **TqSSR046** | TCATGGACAACCCTTGAATG | 59.343 | TGCCTCAAGTGCATAAAAGC | 59.066 |
| **TqSSR047** | TCACACAAGGTGGACGGATA | 59.960 | GCAAACCATTTCCGAGTAGC | 59.713 |
| **TqSSR048** | CGCTTGAGTGTGTTTTCCAA | 59.881 | GTAGGTAGTGGCTCGCTTCG | 60.037 |
| **TqSSR049** | ACGGAATTTTGCAACCAGAT | 59.434 | GTCAACATACACCCTATGAATTGC | 59.677 |
| **TqSSR050** | GGTGGTGGGAGCTCTTGTTA | 60.111 | ATGCAAGACGGCAACTCTCT | 60.020 |

**Continued Supplementary Table S2**

| **Primer** | **Forward primer sequence** | **Tm (℃)** | **Reverse primer sequence** | **Tm (℃)** |
| --- | --- | --- | --- | --- |
| **TqSSR051** | GTCTTCGTTTAATTCGCGTAAG | 58.164 | CCAGAGATGAATGAAATCTGGA | 59.126 |
| **TqSSR052** | CTCGTTCCAAGACGGACATT | 60.111 | ACTTGGAGTATGGGCCACTG | 59.989 |
| **TqSSR053** | GCCGATAGGTGCCGATAGT | 60.073 | CGTCCCAACTAGCTTGTTTCA | 60.290 |
| **TqSSR054** | CGTGCGATGCACGTATAATC | 60.126 | GCCTATTTTGCCCTTCCATT | 60.280 |
| **TqSSR055** | GTAGGTAGTGGCTCGCTTCG | 60.037 | CGCTTGAGTGTGTTTTCCAA | 59.881 |
| **TqSSR056** | TCCAAGATCCAAGTCCAAGG | 60.042 | CGAATTCCGGTGAGTGAGAT | 60.073 |
| **TqSSR057** | GAGATGGACGGAGGGAGTAA | 59.090 | AAAGCCTAGGACAGTCACACAA | 58.912 |
| **TqSSR058** | GGTGCGCTAAATCACCTTGT | 60.140 | TTTCTCTCCGACAAGTGCAG | 59.161 |
| **TqSSR059** | TGGGAAAAGGAAAAGTGACG | 60.081 | TGGATGGAAAGTTAAGGATTGG | 60.177 |
| **TqSSR060** | TGAATCAATTCGACGGTTCA | 60.049 | CGAGAGGAAATTCACCGAAA | 60.184 |
| **TqSSR061** | TTCCTATTTCTCACACCAGGAC | 58.174 | AATTCGATGTGGGACGAAAA | 60.309 |
| **TqSSR062** | GAAAATGGCCTCAAACCAGA | 60.051 | TGGGCATGATGAGCCTAGAT | 60.591 |
| **TqSSR063** | AGGGTTCAGATCGAGGGTTT | 59.935 | CAAAACGTGGACATGCTCAG | 60.301 |
| **TqSSR064** | GGTGTTTCTCATGCAACTCTTC | 58.842 | GCGGTTCGGTTAAGGTTCTT | 60.483 |
| **TqSSR065** | TCGGAAGAAACCAGCAAAAG | 60.357 | CGGAAAGATCAGCTCCACTC | 59.950 |
| **TqSSR066** | GAGCTTCGCACCTCCTAATG | 59.978 | AGCTCGGCACTCTGTCTCTC | 59.890 |
| **TqSSR067** | AATCGAATTGCAACCTCACA | 59.127 | GCATAAATTCCGAGGACGAA | 60.038 |
| **TqSSR068** | CGTCCATCTGTCTCGGATTT | 60.073 | TCGGTTTTCCGTTTCTTGAG | 60.220 |
| **TqSSR069** | TTTTGCCCAGGGATGATTTA | 60.260 | AAGCTCAGGTGCAAATCAGG | 60.397 |
| **TqSSR070** | TGATTCGTGTTGGCATGTTT | 59.972 | CAGCGTGCTTTAGGGTTTTC | 59.883 |
| **TqSSR071** | ACGAATCGACAACGCTACG | 59.879 | TGGAGTGCGTGAGATAAACAA | 59.332 |
| **TqSSR072** | GAGATAGTCGAGATGGAAGAAATGT | 59.211 | GTTCACGTCGCATTGATTTC | 59.131 |
| **TqSSR073** | TGATGAGTTGCGATGGTGAT | 60.080 | GGACGTGGTAGTTATAAGATTGTGG | 60.070 |
| **TqSSR074** | GTGGGAGTTGGAGGACAAAA | 59.943 | AAAAGTCGACCCATCATTCG | 59.933 |
| **TqSSR075** | CACTTTCGGTAAGGCGGTTA | 60.124 | CATGGGACCTTCCACGTAAT | 59.670 |

**Continued Supplementary Table S2**

| **Primer** | **Forward primer sequence** | **Tm (℃)** | **Reverse primer sequence** | **Tm (℃)** |
| --- | --- | --- | --- | --- |
| **TqSSR076** | ATGTGAACGACCACCTCCTC | 59.969 | CCGAGTAAGAAACGGTCCAA | 60.103 |
| **TqSSR077** | CCATGACCTAACGGAAACCA | 60.745 | CCTTCACTTAATGCACCATAGC | 58.789 |
| **TqSSR078** | ACTTCGTCCACCTTGTCCAC | 60.009 | CATGGGAGAAGAGGGCATAA | 60.029 |
| **TqSSR079** | TGGGCAAATAGGGAGACTTG | 60.066 | TCAAGGCTTGCACGTAAGAA | 59.609 |
| **TqSSR080** | ACCGGTGCACGTCTATTTTC | 60.000 | TTCTCGCTCCTCATCAACCT | 59.950 |
| **TqSSR081** | CCCTATGCTCTCCGTTCCTA | 59.288 | CTTTTAGGTAGCCCGTTGTTT | 57.535 |
| **TqSSR082** | CGAGATTCGTATTCGTTTAGGC | 60.115 | TTTCCTGTCTCCCCTTTCAA | 59.641 |
| **TqSSR083** | GGCCAAGTCGGATTTGTTTA | 59.938 | GGTTCGATTCCCACTACCAA | 59.790 |
| **TqSSR084** | CGATTGCTGCCACTAGACAA | 60.011 | GGCTCCTTTGAACGCTGTAG | 60.015 |
| **TqSSR085** | TATGCGCGCAGTTTAGTTTG | 60.038 | GCCTAGTCAAAATGGGAAAGC | 60.089 |
| **TqSSR086** | GATTTGAGTCCATGCCGACT | 60.081 | TCCTTCGTTCCTTCATGACTC | 59.274 |
| **TqSSR087** | ATTGCACCCTCGAGAAATTG | 60.074 | CATGGATGCAAACCAAAGTG | 59.964 |
| **TqSSR088** | GCAATCCAAAGAGGTTGCAT | 60.081 | GAGGCAGTCGAATTCGGTAA | 60.214 |
| **TqSSR089** | GAGAGAGACATGTGGCGTTG | 59.420 | AGAAAGAGAGGATGGGCATT | 57.796 |
| **TqSSR090** | TTGGGTCGTCTCATCATTTG | 59.496 | GCCGAACCATTATGCGTACA | 61.800 |
| **TqSSR091** | CCTATTACCCACCCATGTCG | 60.066 | TCTGATTGGTGTGGATTGGA | 59.893 |
| **TqSSR092** | GGGAGGGGCTACTACCAGAG | 60.088 | CTTTCCCTGTCGAACTGACC | 59.697 |
| **TqSSR093** | AGGCTCGTTAAAATCGGACA | 59.708 | CATCCCCTCGACTCAATGTT | 59.927 |
| **TqSSR094** | CACGCAAGAACCATACCAGA | 59.716 | TCGTTCGAGTCGTTTCTGTG | 60.025 |
| **TqSSR095** | CCACTACGTACGTGATCGAGA | 58.813 | GAGGTTCGATTTCGCCTTTC | 61.072 |
| **TqSSR096** | TGAGCTCAAAGTTCTTCTCCATC | 60.012 | TTGACGGCTCGTGACACTTA | 60.449 |
| **TqSSR097** | ATAGCTCCGGTGTTGGTGTC | 59.997 | GTCAATTGCGCCTCTCTCTC | 60.104 |
| **TqSSR098** | AAATGACACACCACGAACGA | 60.008 | GAGAGCGAGAACAAGCGAGT | 59.898 |
| **TqSSR099** | CCCTCCGCTCTCTTTCTTTC | 60.455 | AGCTGTTCGAGAAGCTCAGG | 59.891 |
| **TqSSR100** | GTGCGACCCCAGTGTTTAGT | 60.035 | CCAAGCCATCATCCATTACC | 60.155 |

**Continued Supplementary Table S2**

| **Primer** | **Forward primer sequence** | **Tm (℃)** | **Reverse primer sequence** | **Tm (℃)** |
| --- | --- | --- | --- | --- |
| **TqSSR101** | ACGTCGTTAGCTGTCCGAGT | 59.937 | AGGCCACTGGACTTGAGAAA | 59.844 |
| **TqSSR102** | GGAAGTGGGGAGAAAGGGTA | 60.298 | GGCCCATTTGATTTTGATGT | 59.628 |
| **TqSSR103** | ATCGATCTTCGAGCAATGGT | 59.658 | CCGCTTGAGAGATGAAGACC | 59.950 |
| **TqSSR104** | GATGCTTGAATGAGCGTCAA | 59.955 | GACACTCCCTCCCTACACCA | 59.962 |
| **TqSSR105** | GTTAGGGCTCCCCTGTTTTC | 59.940 | TGGTCCTACACTTGGCAGAA | 59.288 |
| **TqSSR106** | GTTAGGGCTCCCCTGTTTTC | 59.940 | CAATTCGTGGCCCGTAAATA | 60.691 |
| **TqSSR107** | CCGTACTAGGTTTCGGTGGA | 59.986 | GTCCAAATCCAAATGGCAAA | 60.681 |
| **TqSSR108** | TTGCAAACGCTACTTTGTACG | 59.084 | TGTAATTGTGCTGCGGAAGT | 59.347 |
| **TqSSR109** | GGCCGGCCATAATTTAAC | 57.908 | CGAACTGGAATTTCCTCAACA | 60.096 |
| **TqSSR110** | GGAGAGTTGAGGAGCACTGG | 59.986 | ACCAACGCCTTCTTCCCTAT | 59.962 |
| **TqSSR111** | GCACACGAACCACTCCCTAT | 59.997 | CCCCAAGCTAATGAAACTTGC | 60.962 |
| **TqSSR112** | AAAAGGGGCAAGGACAAAAT | 59.814 | AGGTGAGAAGGGAGGGAGAG | 59.801 |
| **TqSSR113** | AGCCTCATTTGCCTCTTGAA | 59.955 | GCTGATCACCAGCCTCTCTC | 60.104 |
| **TqSSR114** | GCTTTTCATTTCACGGCATT | 60.081 | AGTCAGCCTCCGAACTCTCA | 60.135 |
| **TqSSR115** | ATTCCTTCCCTGCAACTCCT | 60.074 | CTTGTCTCGGGACACCAGTT | 60.151 |
| **TqSSR116** | GAGGAAATATGAATGGGAAGCA | 60.280 | TTCGACAGAACGGAAGGAGT | 59.844 |
| **TqSSR117** | TGTGGGAAGTGGAAGGAAAG | 60.081 | GGCCAGTGCTAAACACGATT | 60.140 |
| **TqSSR118** | GTTCTGCCATTTCCTTGACC | 59.532 | TCGTTGCGGACAATTGATTT | 61.401 |
| **TqSSR119** | GGCAATTGGAACATCAAACA | 59.375 | GGCGCTTTGTTTACACCTCT | 59.383 |
| **TqSSR120** | TCGCAATCGCTGTACTCATC | 59.979 | TATGCGTTTGGGGAAGAGAT | 59.528 |
| **TqSSR121** | GGAGGAAGAGGAGGAAGAGG | 59.367 | AGGTGTGGATTCTCCGTCTC | 59.105 |
| **TqSSR122** | GAGAGAGAGCGAGGGTGTTG | 60.135 | TCACTTGTGCATCCTCATCC | 59.637 |
| **TqSSR123** | CCCACGTTGCTCGAAAGTAT | 60.132 | ATGCTGTTCTCGGTGATTCC | 60.081 |
| **TqSSR124** | GAGAAGAGGAGCAACCAACG | 59.989 | CTCCATCGTCAGCGTCTGT | 59.996 |
| **TqSSR125** | GCGCCTCCATCTTCTTCTCT | 61.010 | AGCCTTAACAATGCACGAGA | 58.529 |

**Continued Supplementary Table S2**

| **Primer** | **Forward primer sequence** | **Tm (℃)** | **Reverse primer sequence** | **Tm (℃)** |
| --- | --- | --- | --- | --- |
| **TqSSR126** | GTAGGTAGTGGCTCGCTTCG | 60.037 | CGCTTGAGTGTGTTTTCCAA | 59.881 |
| **TqSSR127** | CTGGCGGAAATGTAAAGGAA | 60.067 | ATGACCTGCTTTGGGTGACT | 59.579 |
| **TqSSR128** | TTTTAAGCGAGCTTGCACAC | 59.254 | GCCCGTACCCTTGCTTATTT | 60.332 |
| **TqSSR129** | AAGAAATCCAGCTCGCACAG | 60.538 | GGAGCTGAGGCTACCAATCA | 60.362 |
| **TqSSR130** | CTGCATCGAGCCATTGAGTA | 59.972 | GGAAGAGTGAGGTGGCAGAG | 59.986 |
| **TqSSR131** | TTGCGACATGCATCGTAACT | 60.288 | TCGGATCATGCAACAAGAGA | 60.353 |
| **TqSSR132** | ATCGAATGCGAATCTCTCGT | 59.803 | ATCCAGTCCGACGTCGTTTA | 60.517 |
| **TqSSR133** | CGTTAACCCTACCCGCTCTA | 59.244 | CCTTTCTTTCCGTTTCATCG | 59.679 |
| **TqSSR134** | CCACTATGTGTCCTCGCCTA | 58.749 | ACCAAAATGGGGACATAGGA | 59.101 |
| **TqSSR135** | CCCCTTCCATCTTCTTCCTC | 60.008 | CCCCTTCCATCTTCTTCCTC | 60.008 |
| **TqSSR136** | CCGTTAAAAGGGAACTTAATCG | 59.086 | GACAGATCCAACAGTGCAAAA | 58.763 |
| **TqSSR137** | CACCCTTAGCTTCAGCCTTG | 60.008 | GGTACATGGCCCCTGATATG | 60.036 |
| **TqSSR138** | CAAACAAGCAGCAGCAGAAG | 59.928 | GGCTCCTTTGGGGAGATCTAT | 60.769 |
| **TqSSR139** | GTGCCAATTGTTCTGAATGA | 57.081 | ACCGTGAGGCCAGATTAAGA | 59.694 |
| **TqSSR140** | GAGGGAGTGCATACCTTTCG | 59.694 | CCCCTTTCTCACAACACACA | 59.565 |
| **TqSSR141** | CGACGGGTTTCAACTTCTTC | 59.711 | ACAGAGCAGAGGGAGAGCAA | 60.284 |
| **TqSSR142** | ATTGGTCTTGGGCTCCTTTT | 59.940 | TTCGAGGCCCATTTGATAAG | 60.031 |
| **TqSSR143** | TGCATAGCGTGATGCATTTT | 60.245 | AGGCAAGTCACTAGGCAGGA | 60.012 |
| **TqSSR144** | GTCACCTCCGAACCTGAAAA | 60.088 | TCGCCCCTTTCTTTCTCTCT | 60.455 |
| **TqSSR145** | CAATTCGAAAGTTGGGGAAA | 59.910 | CTAGCCACAAGGGCTAGGG | 59.838 |
| **TqSSR146** | AAGCGTCTCAGATCCTCTGC | 59.709 | CCAAGGATACAGAGGGACCA | 59.920 |
| **TqSSR147** | CCCCATGCACTCTTTTCATT | 59.933 | ATCGGAGCTCACTCCACAAG | 60.408 |
| **TqSSR148** | TCCTGCGTATTCTGCATCTG | 59.972 | CTGCGCAAACCTTCAAATC | 59.410 |
| **TqSSR149** | CTGCCCCTCGAGACAATAAA | 60.206 | CCAAAGGTTGGAGGAGTGAG | 59.691 |
| **TqSSR150** | CCAATTTCCGCTAATCCAAA | 59.901 | CTTCCTTCGCACATTTGACA | 59.840 |

**Continued Supplementary Table S2**

| **Primer** | **Forward primer sequence** | **Tm (℃)** | **Reverse primer sequence** | **Tm (℃)** |
| --- | --- | --- | --- | --- |
| **TqSSR151** | CCTTCAAGACGGTGACAACC | 60.545 | CGGGATCGGGAATAGAATTT | 60.109 |
| **TqSSR152** | TGGATTCCGTAAACCCTTTG | 59.795 | TGCTGCTTTCCTTCGTTTCT | 60.132 |
| **TqSSR153** | TTGGAAGGACAACGAGCTTC | 60.375 | CTCAGTTGGGCCCAGTATGT | 59.989 |
| **TqSSR154** | GGGGAGGAGAGAAGAGTGGT | 59.661 | CTCTAGCAATGGCGGAGCTA | 60.637 |
| **TqSSR155** | CATTGGCGAAAGTACGGATT | 59.960 | CGAAATCGCTTCCAAAACAT | 60.074 |
| **TqSSR156** | GAGGCAGTCGAATTCGGTAA | 60.214 | GCAATCCAAAGAGGTTGCAT | 60.081 |
| **TqSSR157** | CAGGCTGTTGCAAGCTATGA | 60.157 | TGGTGGCAAAAGTTCGTTTA | 59.202 |
| **TqSSR158** | TGGTTTTCACGTTTTCACGA | 60.127 | GCATCTCCGTGTTACCCAAA | 60.894 |
| **TqSSR159** | ATGCAAGACGGCAACTCTCT | 60.020 | AGATGAAAGGGGAAGGGAAA | 59.875 |
| **TqSSR160** | CGCTTGAGTGTGTTTTCCAA | 59.881 | GTAGGTAGTGGCTCGCTTCG | 60.037 |
| **TqSSR161** | ACCAACTGCGAAGTTCAGGT | 59.769 | GCAATATCGGCTGCTCTCTC | 60.088 |
| **TqSSR162** | TCTCATAGTTTGGGATGTGTTAGG | 59.433 | CCTAAGGCTTGGAACCGTAA | 59.221 |
| **TqSSR163** | CCAGGGGTTAGGATGAGTGA | 59.920 | GGAGGAAATGAGGAGGGAAG | 60.008 |
| **TqSSR164** | TTGAAGCCGCTCCTACAAGT | 60.015 | CTTCATTCCAGTGCGACTCA | 59.984 |
| **TqSSR165** | TTGGGCTTCAAGCATCCTAC | 60.214 | CCAGCAGAGAGGAGGAGAAG | 59.275 |
| **TqSSR166** | GCTGACGTTTTAGAAACACTAAGGA | 60.218 | GGATTCTTATTCCCCACCAAA | 60.006 |
| **TqSSR167** | GGCATGCAACGTGTAAGAAG | 59.347 | GCCACGTGGGAATTAGAAGA | 60.074 |
| **TqSSR168** | AGGAAGGGTTCATGTTCACG | 59.966 | TAACTGGCATGCTCCACATT | 59.152 |
| **TqSSR169** | TGGGATTTGTATTCCCGTGT | 60.051 | GCCCACTCCGTGAGAAATTA | 60.074 |
| **TqSSR170** | CTACCCTTCCATCACCTCCA | 59.920 | CGACTTCATTCCGCTTCAAT | 60.214 |
| **TqSSR171** | GTGGCCAAATAGCACCACTC | 60.526 | GGATTTTCGCGTGAAGAAGA | 60.331 |
| **TqSSR172** | AAACCCTAACGGGTTCTTGG | 60.214 | TTTCGCTGCAACAAGCAG | 59.852 |
| **TqSSR173** | CAAGAGCCATTTGCAGGTTA | 58.917 | GGAATTTGGCCTTTGATTGA | 59.878 |
| **TqSSR174** | GATGCCCATGAAGAACACCT | 59.934 | TCTCCGTTTCTGGTGGAATC | 60.050 |
| **TqSSR175** | ATTTCCCGACCATTTGATGA | 60.133 | TCAAGCTCGACAGCAAACAC | 60.183 |

**Continued Supplementary Table S2**

| **Primer** | **Forward primer sequence** | **Tm (℃)** | **Reverse primer sequence** | **Tm (℃)** |
| --- | --- | --- | --- | --- |
| **TqSSR176** | CTCAACCAGTCTCGCCTAGC | 60.156 | TCCCACAACGAAATCAATCA | 59.900 |
| **TqSSR177** | GCGAATAAGCTTTGAGTTGCTT | 60.049 | TCTCGTTGCTTTTGTCCACT | 58.497 |
| **TqSSR178** | CATCCAAAGGCAGGCTATTC | 59.668 | AATGCCATCCTTTGAAGCAC | 60.081 |
| **TqSSR179** | GGAAAGCAAACATGGAGAGC | 59.820 | GGAAAAGTCGTCCAATTCCA | 59.910 |
| **TqSSR180** | CCGCTAATTGAGGAGAATGG | 59.662 | TCTGTCCCAATCCCAACTCT | 59.505 |
| **TqSSR181** | TCCTCCCATCTTCATTTTGC | 60.014 | GGCTAAGCACTCACCACACA | 59.905 |
| **TqSSR182** | AAGGCATGATTGCATGTTGA | 60.080 | GCGACATTGAAGATGCTCTG | 59.552 |
| **TqSSR183** | AGACTTGGGGTTTGGGTAGG | 60.218 | GCAAAGCAATGAACGAAAGG | 60.752 |
| **TqSSR184** | GAAGCACTTCATCTCCAAAAGA | 58.579 | CCCTTTTACCTTGCGTTATGG | 60.697 |
| **TqSSR185** | CCCGGAATCAGTTGAAGGTA | 59.926 | ACGAGACGAGAGAGCGAGAA | 60.431 |
| **TqSSR186** | CCATAACAGCTCTGCCACCT | 60.277 | TTTCTGGGATTTTGGAGCTG | 60.184 |
| **TqSSR187** | CTTCACCCGTCATTTTCCAT | 59.790 | CAGCCCCAAGTTTTCATAGC | 59.708 |
| **TqSSR188** | CCCAAACCCAATACCTTCCT | 60.046 | GAGCTCGGCTTCGAGTTAGA | 59.859 |
| **TqSSR189** | CTGCTGAAGCTTGGGGATAG | 59.971 | CACCCCATCTCTCATTTCGT | 59.927 |
| **TqSSR190** | GCATGAATTGCACAAAGTGCT | 61.223 | TGAGCCATGCATGTTTGATT | 60.080 |
| **TqSSR191** | CATCCTCTCCAAAGGTTCCA | 60.042 | TGAAGCTGCTCAAGCAAGAA | 60.011 |
| **TqSSR192** | CAGCCAATCCCTCAAAGAGA | 60.331 | TGTTGGAGCTGACAAATTGC | 59.847 |
| **TqSSR193** | AGATCATGCACCAACTCAACA | 59.154 | TGCTACCGGATTCCAAATTC | 59.901 |
| **TqSSR194** | CTCAAAACAATCACGCCATA | 57.227 | GCAATCCTTTACGTCCTCTTTG | 60.129 |
| **TqSSR195** | TTTTGCCCAACTTCTCATCG | 61.137 | ATGAGTTATGGGCACACACG | 59.445 |
| **TqSSR196** | GGAACTGGAAACCATGTCGT | 59.827 | TGTACTCCCATGCTGAATCG | 59.673 |
| **TqSSR197** | GCCCGTACCAGTCAAGTGAT | 59.997 | CGATAGCGGACTGGATGAGT | 60.240 |
| **TqSSR198** | TTGTGAGGGATTATGTTTGGAG | 58.949 | TCAACCTTATCTCCTCTTAACCTTC | 58.481 |
| **TqSSR199** | AATTTGCCCACGGTGAGTAG | 59.993 | ATGCACACCCAATATCCACA | 59.654 |
| **TqSSR200** | AGGAGGAGGAGGGAAGATGA | 60.149 | TGTGGGTATTGTGGCAGAGA | 60.112 |

**Continued Supplementary Table S2**

| **Primer** | **Forward primer sequence** | **Tm (℃)** | **Reverse primer sequence** | **Tm (℃)** |
| --- | --- | --- | --- | --- |
| **TqSSR201** | CACCGTTTGGGAAAAGTCAT | 59.830 | TCCTACGCGCAATAATCCTC | 60.196 |
| **TqSSR202** | TGATTGTTTCGCTCGTGTTC | 59.847 | CATCCCACGCTCTCTTTCTC | 59.950 |
| **TqSSR203** | TTGAGCTCCTTCCCAAAATG | 60.184 | GCTTTCGTTTCATTCCTCAA | 57.944 |
| **TqSSR204** | TTTGGTCCGACTGTTCACAC | 59.571 | CGGTAAAATCGAACAAGGGTA | 58.997 |
| **TqSSR205** | GCCAATGACTTTGGGTCAGT | 59.973 | TACCGATGGAAATCCCAAAA | 60.124 |
| **TqSSR206** | TACCCCACCCTCCTCTCTCT | 60.066 | CGGTTTTGAGGTGCTTGATT | 60.110 |
| **TqSSR207** | AGCGTAGAGCTGGGAAATGA | 59.978 | CGTGGCGATTGATATGATTG | 59.914 |
| **TqSSR208** | TTCAGTGAGTGATCGGCTGT | 59.420 | TCAAGATGGCTTTCAGAGCA | 59.673 |
| **TqSSR209** | ATGTGGCGGAACTTTGTTTT | 59.476 | CGGTGATGAAAAGAGCACAA | 59.840 |
| **TqSSR210** | ACCAAGTTGAGAAGGGTCCA | 59.549 | TAATGCGTGCCTGGATTACA | 60.096 |
| **TqSSR211** | TTTTCTGCCATTCTCCTTGG | 60.184 | TCCCATCCAACATCAACAAA | 59.750 |
| **TqSSR212** | AAGAACCAATCTAGACCTTCCATC | 59.073 | CAAGAAGACGAAGAAGAAGAAGAAG | 59.404 |
| **TqSSR213** | TGCTCCTATAAGCTCGTTTGTG | 59.558 | CCATCTCACCTTTTCAACGAA | 60.096 |
| **TqSSR214** | CATGTTCGTCGGAAATTGTG | 59.964 | TGCAATTTAGGCGGGAAATA | 60.405 |
| **TqSSR215** | CGTGAGGCCGGATTAAGAT | 60.044 | GTTCTGAATGAAAGTGCCAATC | 58.697 |
| **TqSSR216** | GGACCATTGATCTGTGTGATCT | 58.871 | CCCGTGGACGTAGATCATTT | 59.813 |
| **TqSSR217** | TCGAATCCGCATTGCTATTA | 59.266 | TCCCTATGCTCTCCGTTCAT | 59.653 |
| **TqSSR218** | TATTCGACAACCACGCTCAG | 59.864 | CAATCGAGAGCTCGACATGA | 60.096 |
| **TqSSR219** | CAGCGTCTGTTGCTAGGTTG | 59.658 | AAGCCCGTTGGTAGTCAATG | 59.993 |
| **TqSSR220** | AACGGCGAGAATTAGGGTTT | 59.966 | CGTGTTGGCAATTTGATACG | 59.992 |
| **TqSSR221** | TGGGATTCAACGGAACATTT | 60.170 | GGTTCTGAATGAGGGGATTG | 59.336 |
| **TqSSR222** | GAAGAAGAGCCGAGCCTACA | 59.717 | TACATCTCCAGCCCTTCCAC | 60.073 |
| **TqSSR223** | TGCAACTGATCCAATCCGTA | 60.073 | TCACTCCTCCTTTTGCTTGG | 60.366 |
| **TqSSR224** | CATCTCTCTCTCGCAGCTCA | 59.551 | AAGGAAGCTCGGGAAACAAT | 60.074 |
| **TqSSR225** | CAAGACGGTGACAACCAATG | 60.001 | CGGGATTTCGGGTAGGTACT | 60.200 |

**Continued Supplementary Table S2**

| **Primer** | **Forward primer sequence** | **Tm (℃)** | **Reverse primer sequence** | **Tm (℃)** |
| --- | --- | --- | --- | --- |
| **TqSSR226** | ACGGTGGTCTACGTGGAGAG | 60.174 | GGGAAAAGGTTGCAACAGAA | 60.088 |
| **TqSSR227** | GAAGTGGAAAATCCACGTTTG | 59.462 | GGTGAGTACTAAGAACCAATCTCCA | 59.947 |
| **TqSSR228** | AGAATCGTACGGGCAACATT | 59.457 | TCTTTCCTCCTTGCCACTTG | 60.366 |
| **TqSSR229** | CAGCTCCATTGTTGCATGAT | 59.679 | GGTTGTGTCTCTTCGCCATT | 60.119 |
| **TqSSR230** | CCTCAAACGGTGGAGAAGAT | 59.137 | CCTCTGCTTCTGCTCGATTC | 60.240 |
| **TqSSR231** | GGGTTTTGGTCAAATCATGG | 60.029 | TAAACGGGTGTGATGGGTTC | 60.615 |
| **TqSSR232** | GCTTTTCCGATTATGGAGCA | 60.175 | TGGTGATATGATTGCGAGGA | 60.033 |
| **TqSSR233** | TGCATGGACACGGTAAGAGA | 60.263 | AATTGGGTCCCACCACTTTT | 60.455 |
| **TqSSR234** | GGATCGATGGGTACGTGTTT | 59.676 | TCAAATGTCCCATGAAGTTCC | 59.782 |
| **TqSSR235** | GGCAAAGGAGAAGCTGTTTG | 59.993 | AGTGGATGCTCTCGCTTTGT | 60.020 |
| **TqSSR236** | AATGTGGGTGTGTTTGGTTG | 59.161 | CCACATACCACCGTGAAACA | 60.278 |
| **TqSSR237** | AGTGCATGGTTTTCACACGA | 60.160 | TCTTTCTTTGGCGATTCTGG | 60.323 |
| **TqSSR238** | TTCTCCAGCAAATGTGACAA | 57.806 | GTGAGGCCGGATTAAGATTG | 59.528 |
| **TqSSR239** | CAACAACCCCAACAGGAAGT | 59.861 | GGCGAGGTGCTCTCTCTCTA | 59.851 |
| **TqSSR240** | TGGACTAATATGATGGGATGTAGG | 59.145 | ATTTCGCACCAAAGAGAACG | 60.249 |
| **TqSSR241** | TTTGCTTCGGTGGACAAAA | 60.219 | CTCCGAACTCATCTCCCAGA | 60.340 |
| **TqSSR242** | CTCATAGTTAAATGGGACGAAAGG | 60.238 | GGCCCGTGACACAATTCTA | 59.512 |
| **TqSSR243** | CAAGAAAGGTTGGACCTGGA | 60.081 | TGGGCTCTTGGGATAACAAC | 59.933 |
| **TqSSR244** | CTAGGGTTCAGCCGTTCTTG | 59.869 | CACCTCATCGACGCAAACTA | 59.864 |
| **TqSSR245** | TGGGAACATGTGAAGTCCAA | 59.935 | TCAATGGACATCGAAACCAA | 59.900 |
| **TqSSR246** | AATCCATACCCACCATCACG | 60.461 | TTCTCGACGGCTACAATTCC | 60.214 |
| **TqSSR247** | AATCCATACCCACCATCACG | 60.461 | ACCGGGAAAGTCAACTCCTT | 59.972 |
| **TqSSR248** | TTGCACAACCACCGATATTT | 58.895 | AATTTGGAGGCAATGAGCAC | 60.081 |
| **TqSSR249** | TGAGCAGCTCGACTCGTTTA | 59.888 | TCTTCAGGTGCCTCTGTAACC | 59.343 |
| **TqSSR250** | CTCGCAAGCCTTTCCTAAAC | 59.111 | GAAAGGAGACGCTCGAGAAA | 59.694 |

**Continued Supplementary Table S2**

| **Primer** | **Forward primer sequence** | **Tm (℃)** | **Reverse primer sequence** | **Tm (℃)** |
| --- | --- | --- | --- | --- |
| **TqSSR251** | CGGATTTACAACTGCCATTGA | 60.871 | GGCCCAACAACACACCTAAT | 59.717 |
| **TqSSR252** | TGCATTATGATGGTTGATGC | 57.928 | AAACGACGGACGATTGCTAC | 60.140 |
| **TqSSR253** | TTTTACGGGGAGTGTTGGAG | 59.964 | AGATGGGAAGAGATGGCCTA | 58.691 |
| **TqSSR254** | TTCCCTCTCCCTCTCCATCT | 60.149 | CATGGATGGACAGATCATGC | 59.882 |
| **TqSSR255** | CCCATGAATTGTGGAACAAA | 59.222 | CAGATCCCGTATTTGTTGCTTT | 60.364 |
| **TqSSR256** | GGACGTAGGAGGGTTTCTCC | 59.935 | GTTAGGCCAATGCAGGTTGT | 60.000 |
| **TqSSR257** | GGGGCCATGAATATTTTGGTA | 60.737 | ATCGTGCATCGGATGTTGTA | 59.955 |
| **TqSSR258** | TGGGTGATTAATCCCTTGAA | 57.860 | GCATATAACGCATTGTGCAT | 57.164 |
| **TqSSR259** | CACACAGATCAATGGTCCAGA | 59.535 | TGCAGATTTTGACACTGATGG | 59.707 |
| **TqSSR260** | TGCGGATAGTGAGCTCTTCA | 59.697 | TGTCCTTCCCGTCTTACCAC | 59.966 |
| **TqSSR261** | TTATGCACGCGTAGTGTTCC | 59.759 | CGGAACCAACATTTCCTCAT | 59.790 |
| **TqSSR262** | TAGACCCTAGGCCAAACGAA | 59.702 | TTGTAAACCACCCGAACACA | 59.857 |
| **TqSSR263** | GACGATGAGCTCGGAACAAT | 60.226 | TAGGAAAATCGGGGTGTGAC | 59.790 |
| **TqSSR264** | CCGAATTAAACGGGTACGTG | 60.236 | TGGTTGGTTTGAAGAAGGGTA | 59.457 |
| **TqSSR265** | TCTCTCCGCAACCCTAGAAA | 59.948 | ATGGTTCAACTTTCGGTTCG | 59.971 |
| **TqSSR266** | CAACCCGGATCTATCAGGAA | 59.887 | AATTTTGCGTCCAGCCTTTA | 59.720 |
| **TqSSR267** | TCGTCGACTGCATTGTTAGC | 60.019 | TGCTTCATCAACTTGGTTCG | 59.840 |
| **TqSSR268** | TGTGGTATGCATCCTCCGTA | 59.948 | TGCTCCCCTATCAATCCATC | 59.854 |
| **TqSSR269** | AGTGGCGACAATCTTGGAAC | 60.119 | ATCTTGTCCGCTTGTTTTGG | 60.110 |
| **TqSSR270** | GAGATTTGACAATTCGGTTCG | 59.560 | TCAAACGGCTCCTTAATGCT | 59.845 |
| **TqSSR271** | TGACAATTCGCTTCGCATAA | 60.356 | AATGGTGGGAATCATTGGAA | 59.991 |
| **TqSSR272** | AACCATCGGCTCGGTTAAG | 60.081 | GGTGTTCCTCATGCAACTCTT | 59.201 |
| **TqSSR273** | CCTAACTAACGCAGGTAGCTTT | 57.063 | CGCTTCCTATGCTCTTCGTT | 59.615 |
| **TqSSR274** | CACTGCCAGGGGTTAGGATA | 59.948 | TGCAGTGATGAAGGCAGAAC | 59.992 |
| **TqSSR275** | TCAATTGGTCAGGTCGATGA | 60.048 | TGAGGCTGATACCCAGTCAA | 59.241 |

**Continued Supplementary Table S2**

| **Primer** | **Forward primer sequence** | **Tm (℃)** | **Reverse primer sequence** | **Tm (℃)** |
| --- | --- | --- | --- | --- |
| **TqSSR276** | CGTGTAATGCAGGTTGATGC | 60.142 | TAAGGGTCACGACCCAAAAC | 59.830 |
| **TqSSR277** | TGGATAAGGCTGGTTTGTTG | 58.623 | ATCCCATGATTTGAGGGACA | 60.135 |
| **TqSSR278** | TTTCACGGCCGAAAATAATC | 59.907 | CTCATGAACACGGTTGATCCT | 59.981 |
| **TqSSR279** | ATTTAGGGCCCGTTTGGTAT | 59.572 | ATGCGTTTGGTGCAAATTGT | 61.323 |
| **TqSSR280** | ATATTCGATTGGTGCGGTGT | 60.221 | CCCGAATGACTTTTCACAAGA | 60.096 |
| **TqSSR281** | GGAGGGTTTAGAGCGAACCT | 59.713 | AGAGCTTCCGGGTAGAGGAG | 59.972 |
| **TqSSR282** | GGAATTTGTGCTGTCGCTTT | 60.257 | AGCAAGCGAAGCGAGTCTAC | 59.927 |
| **TqSSR283** | TGCGGAGCAGGTTAGTTAATG | 60.271 | GGCGGGAGTATATTGACACA | 58.450 |
| **TqSSR284** | TTTGTAACCGGTTCGTGTGA | 60.004 | CGTTACCTGCGGATTCATTT | 59.960 |
| **TqSSR285** | GTCGCCCCATCAAGAGAATA | 60.036 | GGCAGCCCAAGAAATATGAA | 60.038 |
| **TqSSR286** | TCCAGTCTCGAAAATGTGGA | 59.215 | CTGAATCGCAGGTTTCGTTT | 60.249 |
| **TqSSR287** | CGAACCACGAACTGGAATTT | 59.971 | CGCTTGATCAACACAAATGG | 60.111 |
| **TqSSR288** | CCAATCTCCTCATCCAGGAA | 60.003 | GCAGCAGCAGATGAACAAAA | 60.142 |
| **TqSSR289** | TGGGAGCGATGATTAGAACC | 60.036 | CGGCTCGGGACACTTATTTA | 60.089 |
| **TqSSR290** | ACACGTGGACAAGGGAGAAG | 60.151 | ACAAATATACGGCGGAGACG | 59.982 |
| **TqSSR291** | GGTGGGGAGGTTGTTCATAA | 59.647 | TACTGCCGCTTCAAACTCCT | 60.015 |
| **TqSSR292** | TTCTCACTCTCTCCGGCAAT | 59.950 | GCAGAAATCACGACTCACGA | 59.992 |
| **TqSSR293** | CAGCGTGCTTTAGGGTTTTC | 59.883 | CTGCGTTTGCTGACTGGATA | 60.011 |
| **TqSSR294** | GGTGTTCCTCATGCAACTCTT | 59.201 | CCTAAGGCTTGGAACCGTAA | 59.221 |
| **TqSSR295** | GGTGTTCCTCATGCAACTCTT | 59.201 | AGGCTTGAAACCGTAACTGG | 59.238 |
| **TqSSR296** | CTCCTTCCCTTGTGCTTGTC | 59.844 | CATGGGGAAATAACGAGCAT | 59.784 |
| **TqSSR297** | TAGGCGTTCTCATTCGGAAG | 60.344 | CCGTCTTCTCCATCTTCGTC | 59.803 |
| **TqSSR298** | TGGGTATCTTGGGAGTTGTTT | 58.423 | CAACTCATTCTCAAGTGTCAACC | 58.758 |
| **TqSSR299** | TGAAGAAACACGCATGAACC | 59.697 | CTTCGTTCGTTCGAGTCGTT | 60.431 |
| **TqSSR300** | AGCCACCAGATCAGCAGTTT | 59.874 | AGAGAGGTCGCTTGAAACCA | 59.989 |
