# Supplementary material for: Creation of new germplasm resources, development of SSR markers, and screening of monoterpene synthases in thyme
Source: BMC Plant Biol. 2023 Jan 6;23:13. doi: 10.1186/s12870-022-04029-2 (PMC9817278; doi:10.1186/s12870-022-04029-2)
Supplement: Supplementary file 5 — Additional file 5: Supplementary Table S3. SSR primers used for the identification of F1 hybrids in the Tl × Tvf population. [file 12870_2022_4029_MOESM5_ESM.docx]

**Supplementary Table S3 SSR primers used for the identification of F_1_ hybrids in the Tl × Tvf population.**

| **Primer** | **Forward primer sequence** | **Tm (℃)** | **Reverse primer sequence** | **Tm (℃)** |
| --- | --- | --- | --- | --- |
| **TqSSR107** | CCGTACTAGGTTTCGGTGGA | 59.986 | GTCCAAATCCAAATGGCAAA | 60.681 |
| **TqSSR108** | TTGCAAACGCTACTTTGTACG | 59.084 | TGTAATTGTGCTGCGGAAGT | 59.347 |
| **TqSSR119** | GGCAATTGGAACATCAAACA | 59.375 | GGCGCTTTGTTTACACCTCT | 59.383 |
| **TqSSR124** | GAGAAGAGGAGCAACCAACG | 59.989 | CTCCATCGTCAGCGTCTGT | 59.996 |
| **TqSSR128** | TTTTAAGCGAGCTTGCACAC | 59.254 | GCCCGTACCCTTGCTTATTT | 60.332 |
| **TqSSR130** | CTGCATCGAGCCATTGAGTA | 59.972 | GGAAGAGTGAGGTGGCAGAG | 59.986 |
| **TqSSR142** | ATTGGTCTTGGGCTCCTTTT | 59.94 | TTCGAGGCCCATTTGATAAG | 60.031 |
| **TqSSR146** | AAGCGTCTCAGATCCTCTGC | 59.709 | CCAAGGATACAGAGGGACCA | 59.92 |
| **TqSSR187** | CTTCACCCGTCATTTTCCAT | 59.79 | CAGCCCCAAGTTTTCATAGC | 59.708 |
| **TqSSR192** | CAGCCAATCCCTCAAAGAGA | 60.331 | TGTTGGAGCTGACAAATTGC | 59.847 |
| **TqSSR194** | CTCAAAACAATCACGCCATA | 57.227 | GCAATCCTTTACGTCCTCTTTG | 60.129 |
| **TqSSR217** | TCGAATCCGCATTGCTATTA | 59.266 | TCCCTATGCTCTCCGTTCAT | 59.653 |
| **TqSSR248** | TTGCACAACCACCGATATTT | 58.895 | AATTTGGAGGCAATGAGCAC | 60.081 |
| **TqSSR271** | TGACAATTCGCTTCGCATAA | 60.356 | AATGGTGGGAATCATTGGAA | 59.991 |
| **TqSSR285** | GTCGCCCCATCAAGAGAATA | 60.036 | GGCAGCCCAAGAAATATGAA | 60.038 |
| **TqSSR289** | TGGGAGCGATGATTAGAACC | 60.036 | CGGCTCGGGACACTTATTTA | 60.089 |
| **TqSSR290** | ACACGTGGACAAGGGAGAAG | 60.151 | ACAAATATACGGCGGAGACG | 59.982 |
| **TqSSR292** | TTCTCACTCTCTCCGGCAAT | 59.95 | GCAGAAATCACGACTCACGA | 59.992 |
