# Supplementary material for: Creation of new germplasm resources, development of SSR markers, and screening of monoterpene synthases in thyme
Source: BMC Plant Biol. 2023 Jan 6;23:13. doi: 10.1186/s12870-022-04029-2 (PMC9817278; doi:10.1186/s12870-022-04029-2)
Supplement: Supplementary file 6 — Additional file 6: Supplementary Table S4. SSR primers used for the identification of F1 hybrids in the Tve × Tq population. [file 12870_2022_4029_MOESM6_ESM.docx]

**Supplementary Table S4. SSR primers used for the identification of F_1_ hybrids in the Tve × Tq population.**

| **Primer** | **Forward primer sequence** | **Tm (℃)** | **Reverse primer sequence** | **Tm (℃)** |
| --- | --- | --- | --- | --- |
| **TqSSR001** | CTTCTCCTCCCCGTCAATCT | 60.586 | GAGGGAAGGGAGAAGAATCG | 60.147 |
| **TqSSR004** | TGATTGGGGTTGTGAAGACA | 59.935 | TGTTGTGTGTTTTGCTGCTG | 59.504 |
| **TqSSR028** | ATTCAGGCGTGGGATATCAA | 60.296 | CCATACCATGTGTTTGGCATA | 59.172 |
| **TqSSR034** | GCATCAAAGAGAACGCAATC | 58.451 | CCGTGGAAATCCTTCCTCTA | 59.122 |
| **TqSSR041** | TGTAATGCCCACTCCGTGTA | 59.988 | ACGCCCAATTCACAAAATTC | 59.807 |
| **TqSSR056** | TCCAAGATCCAAGTCCAAGG | 60.042 | CGAATTCCGGTGAGTGAGAT | 60.073 |
| **TqSSR078** | ACTTCGTCCACCTTGTCCAC | 60.009 | CATGGGAGAAGAGGGCATAA | 60.029 |
| **TqSSR104** | GATGCTTGAATGAGCGTCAA | 59.955 | GACACTCCCTCCCTACACCA | 59.962 |
| **TqSSR108** | TTGCAAACGCTACTTTGTACG | 59.084 | TGTAATTGTGCTGCGGAAGT | 59.347 |
| **TqSSR128** | TTTTAAGCGAGCTTGCACAC | 59.254 | GCCCGTACCCTTGCTTATTT | 60.332 |
| **TqSSR131** | TTGCGACATGCATCGTAACT | 60.288 | TCGGATCATGCAACAAGAGA | 60.353 |
| **TqSSR142** | ATTGGTCTTGGGCTCCTTTT | 59.94 | TTCGAGGCCCATTTGATAAG | 60.031 |
| **TqSSR146** | AAGCGTCTCAGATCCTCTGC | 59.709 | CCAAGGATACAGAGGGACCA | 59.92 |
| **TqSSR170** | CTACCCTTCCATCACCTCCA | 59.92 | CGACTTCATTCCGCTTCAAT | 60.214 |
| **TqSSR179** | GGAAAGCAAACATGGAGAGC | 59.82 | GGAAAAGTCGTCCAATTCCA | 59.91 |
| **TqSSR182** | AAGGCATGATTGCATGTTGA | 60.08 | GCGACATTGAAGATGCTCTG | 59.552 |
| **TqSSR194** | CTCAAAACAATCACGCCATA | 57.227 | GCAATCCTTTACGTCCTCTTTG | 60.129 |
| **TqSSR195** | TTTTGCCCAACTTCTCATCG | 61.137 | ATGAGTTATGGGCACACACG | 59.445 |
| **TqSSR209** | ATGTGGCGGAACTTTGTTTT | 59.476 | CGGTGATGAAAAGAGCACAA | 59.84 |
| **TqSSR225** | CAAGACGGTGACAACCAATG | 60.001 | CGGGATTTCGGGTAGGTACT | 60.2 |
| **TqSSR257** | GGGGCCATGAATATTTTGGTA | 60.737 | ATCGTGCATCGGATGTTGTA | 59.955 |
| **TqSSR269** | AGTGGCGACAATCTTGGAAC | 60.119 | ATCTTGTCCGCTTGTTTTGG | 60.11 |
| **TqSSR284** | TTTGTAACCGGTTCGTGTGA | 60.004 | CGTTACCTGCGGATTCATTT | 59.96 |
