# Supplementary material for: Creation of new germplasm resources, development of SSR markers, and screening of monoterpene synthases in thyme
Source: BMC Plant Biol. 2023 Jan 6;23:13. doi: 10.1186/s12870-022-04029-2 (PMC9817278; doi:10.1186/s12870-022-04029-2)
Supplement: Supplementary file 7 — Additional file 7: Supplementary Table S5. Relative contents of volatile organic compounds (VOCs) in the leaves of Tl, Tvf, and their F1 progeny. [file 12870_2022_4029_MOESM7_ESM.docx]

**Supplementary Table S5 Relative contents of volatile organic compounds (VOCs) in the leaves of Tl, Tvf, and their F_1_ progeny.**

| **No.** | **VOC** | **RI Cal^a^** | **RI Lit^b^** | **Relative content (%)^c^** | | | | | | | | | | | | | | | |
| --- | --- | --- | --- | --- | --- | --- | --- | --- | --- | --- | --- | --- | --- | --- | --- | --- | --- | --- | --- |
|  |  |  |  | **Tl** | **Tvf** | **F_1_-1** | **F_1_-2** | **F_1_-3** | **F_1_-4** | **F_1_-5** | **F_1_-6** | **F_1_-7** | **F_1_-8** | **F_1_-9** | **F_1_-10** | **F_1_-11** | **F_1_-12** | **F_1_-13** | **F_1_-14** |
| **1** | Thymol | 1,292 | 1,291 | - | - | 12.51 ± 1.18a | 8.03 ± 0.88c | 0.27 ± 0.05d | - | 0.61 ± 0.06d | - | - | 9.55 ± 1.16b | 8.39 ± 1.56c | 0.73 ± 0.15d | - | - | - | - |
| **2** | Carvacrol | 1,303 | 1,299 | - | - | 13.93 ± 3.96a | 9.72 ± 1.04b | 0.38 ± 0.04c | - | 0.89 ± 0.10c | - | - | 12.17 ± 1.22a | 12.92 ± 1.85a | - | - | - | - | - |
| **3** | *p*-Cymene | 1,024 | 1,025 | - | - | 16.52 ± 2.74c | 23.22 ± 4.04b | - | - | 0.92 ± 0.02d | - | - | 29.39 ± 4.81a | 23.01 ± 2.73b | 0.51 ± 0.07d | - | - | - | - |
| **4** | γ-Terpinene | 1,059 | 1,060 | - | 2.04 ± 0.10d | 14.16 ± 1.80a | 9.50± 1.02c | - | - | - | - | - | 9.02 ± 0.81c | 11.36 ± 1.81b | - | - | 9.21 ± 0.24c | - | - |
| **5** | Geraniol | 1,256 | 1,255 | 22.75 ± 4.41b | - | - | - | 18.17 ± 2.28c | 22.75 ± 0.76b | 23.79 ± 0.02b | 28.98 ± 1.40a | 4.48 ± 0.02d | - | - | 28.84 ± 0.03a | 26.91 ± 6.57b | - | 25.44 ± 1.07ab | 23.57 ± 1.30b |
| **6** | Geraniol acetate | 1,386 | 1,382 | 41.75 ± 1.26a | - | - | - | 15.76 ± 1.91e | 29.59 ± 2.99b | 21.89 ± 0.03d | 23.41 ± 4.43cd | 27.75 ± 0.03bc | - | - | 15.05 ± 1.69e | 23.60 ± 1.59d | - | 22.50 ± 2.22d | 24.56 ± 0.46cd |
| **7** | Cis-Geraniol | 1,227 | 1,228 | 1.09 ± 0.27f | - | - | - | 2.85 ± 0.44c | 2.02 ± 0.06e | 2.51 ± 0.02cd | 2.16 ± 0.33de | 0.67 ± 0.02f | - | - | 2.83 ± 0.38c | 4.39 ± 0.49a | - | 3.56 ± 0.43b | 3.56 ± 0.37b |
| **8** | α-Terpineol | 1,191 | 1,189 | 11.76 ± 0.36a | - | - | - | - | - | - | - | 3.00 ± 0.02c | - | 0.31 ± 0.01d | - | - | 9.07 ± 0.86b | - |  |
| **9** | α-Terpineol acetate | 1,354 | 1,350 | - | 61.37 ± 3.52a | 0.51 ± 0.04d | - | - | - | - | - | 28.03 ± 0.03c | - | - | - | - | 57.44 ± 3.50b | - | - |

**Continued Supplementary Table S5**

| **No.** | **VOC** | **RI Cal^a^** | **RI Lit^b^** | **Relative content (%)^c^** | | | | | | | | | | | | | | | |
| --- | --- | --- | --- | --- | --- | --- | --- | --- | --- | --- | --- | --- | --- | --- | --- | --- | --- | --- | --- |
|  |  |  |  | **Tl** | **Tvf** | **F_1_-1** | **F_1_-2** | **F_1_-3** | **F_1_-4** | **F_1_-5** | **F_1_-6** | **F_1_-7** | **F_1_-8** | **F_1_-9** | **F_1_-10** | **F_1_-11** | **F_1_-12** | **F_1_-13** | **F_1_-14** |
| **10** | Linalool | 1,099 | 1,099 | 1.14 ± 0.34bc | 1.29 ± 0.08b | 1.59 ± 0.07a | 1.54 ± 0.09a | 0.93 ± 0.04de | 0.76 ± 0.09efg | 1.02 ± 0.03cd | 0.82 ± 0.07ef | 0.58 ± 0.02gh | 1.57 ± 0.08a | 1.70 ± 0.17a | 0.64 ± 0.04fgh | 0.63 ± 0.02fgh | 0.67 ± 0.05fgh | 0.75 ± 0.04efg | 0.54 ± 0.07h |
| **11** | Eucalyptol | 1,030 | 1,032 | 1.25 ± 0.24b | 4.54 ± 1.87a | 4.03 ± 0.48a | 5.07 ± 0.54a | 0.95 ± 0.10b | 5.16 ± 1.18a | - | 4.58 ± 0.61a | - | - | 4.45 ± 0.86a | 3.92 ± 1.06a | 0.74 ± 0.17b | - | 0.99 ± 0.12b | 3.93 ± 0.26a |
| **12** | β-Caryophyllen | 1,420 | 1,419 | 4.13 ± 0.48def | 4.74 ± 0.13def | 3.5 ± 1.26ef | 11.1 ± 0.46b | 15.51 ± 2.62a | 7.52 ± 1.74c | 11.37 ± 0.01b | 5.04 ± 1.19def | 2.71 ± 0.02f | 6.32 ± 0.74cd | 5.96 ± 0.59cd | 7.71 ± 0.48c | 12.31 ± 3.37b | 5.37 ± 0.83cde | 12.06 ± 0.57b | 6.35 ± 0.62cd |
| **13** | Endo-Borneol | 1,164 | 1,167 | 1.90 ± 0.31ef | - | 1.96 ± 0.46ef | 3.95 ± 0.31b | 5.05 ± 0.31a | 1.97 ± 0.13ef | 3.03 ± 0.02c | 4.37 ± 0.65b | 0.51 ± 0.02g | 2.21 ± 0.14ef | 1.68 ± 0.68f | 2.44 ± 0.10de | 2.18 ± 0.20ef | - | 2.77 ± 0.08cd | 3.91 ± 0.07b |
| **14** | Bornyl acetate | 1,286 | 1,285 | 2.05 ± 0.74cd | 1.54 ± 0.41ef | - | - | 2.97 ± 0.22b | 2.22 ± 0.12c | 1.70 ± 0.01de | 1.69 ± 0.23de | 3.68 ± 0.03a | - | - | 0.60 ± 0.04h | 0.97 ± 0.04gh | - | 1.23 ± 0.08fg | 1.71 ± 0.09de |
| **15** | β-Bisabolene | 1,058 | 1,059 | 6.52 ± 0.98ab | - | 5.28 ± 1.53b | 0.78 ± 0.05de | - | 6.36 ± 1.19ab | - | 1.10 ± 0.18de | 3.39 ± 0.02c | 1.48 ± 0.19d | 6.78 ± 1.47a | - | 1.39 ± 0.17d | 7.46 ± 0.96a | 1.64 ± 0.40d | 1.14 ± 0.12de |
| **16** | α-Citra | 1,271 | 1,270 | 3.52 ± 0.58b | 0.51 ± 0.01e | - | - | 4.01 ± 0.37a | 0.86 ± 0.22e | 4.25 ± 0.02a | 1.62 ± 0.18cd | 1.99 ± 0.03c | - | - | 1.45 ± 0.19d | 2.05 ± 0.46c | - | 1.48 ± 0.22d | 1.80 ± 0.47cd |
| **17** | Germacrene D | 1,482 | 1,481 | 0.52 ± 0.47h | 4.49 ± 0.49abcd | 5.09 ± 1.82ab | 5.96 ± 0.99a | 3.26 ± 0.11def | 2.03 ± 0.50fg | 4.83 ± 0.02abc | 3.17 ± 0.83def | 0.45 ± 0.03h | 5.73 ± 1.06a | 3.57 ± 0.68cdef | 2.81 ± 0.61ef | 3.74 ± 1.33bcde | 0.90 ± 0.23gh | 2.90 ± 0.24ef | 3.27 ± 0.35def |

^a^ RI Cal, calculated according to C7–C40.

^b^ RI Lit, obtained by searching the mass spectrum database NIST v14.0.

^c^ Tl, *T. longicaulis*; Tvf, *T. vulgaris* ‘Fragrantissimus’.
