# Supplementary material for: Creation of new germplasm resources, development of SSR markers, and screening of monoterpene synthases in thyme
Source: BMC Plant Biol. 2023 Jan 6;23:13. doi: 10.1186/s12870-022-04029-2 (PMC9817278; doi:10.1186/s12870-022-04029-2)
Supplement: Supplementary file 8 — Additional file 8: Supplementary Table S6. Relative contents of VOCs in the leaves of Tve, Tq, and their F1 progeny. [file 12870_2022_4029_MOESM8_ESM.docx]

**Supplementary Table S6 Relative contents of VOCs in the leaves of Tve, Tq, and their F_1_ progeny.**

| **No.** | **VOC** | **RI Cal^a^** | **RI Lit^b^** | **Relative content (%)^c^** | | | | | | | | | | | | |
| --- | --- | --- | --- | --- | --- | --- | --- | --- | --- | --- | --- | --- | --- | --- | --- | --- |
|  |  |  |  | **Tq** | **Tve** | **F_1_-1** | **F_1_-2** | **F_1_-3** | **F_1_-4** | **F_1_-5** | **F_1_-6** | **F_1_-7** | **F_1_-8** | **F_1_-9** | **F_1_-10** | **F_1_-11** |
| **1** | *p*-Cymene | 1,024 | 1,025 | 23.00 ± 0.09c | 18.42 ± 0.12d | - | - | 37.42 ± 1.92a | 27.03 ± 2.67b | 23.16 ± 2.39c | - | 1.11 ± 0.12e | 18.56 ± 0.51d | - | - | 21.14 ± 0.63c |
| **2** | γ-Terpinene | 1,058 | 1,060 | 11.45 ± 0.07c | 13.96 ± 0.08b | - | - | 17.14 ± 0.71a | 9.03 ± 1.68d | 8.55 ± 1.78d | - | 0.72 ± 0.06f | 12.58 ± 1.17bc | - | - | 6.85 ± 0.62e |
| **3** | Thymol | 1,293 | 1,291 | 2.12 ± 0.03d | 35.43 ± 0.14b | - | - | 23.57 ± 0.79c | 24.71 ± 2.20c | 46.19 ± 2.76a | - | - | 46.38 ± 1.89a | - | - | 33.26 ± 1.39b |
| **4** | Carvacrol | 1,301 | 1,299 | 20.74 ± 0.07a | 2.27 ± 0.05b | - | - | 1.64 ± 0.22c | - | 2.10 ± 0.17b | - | - | 0.71 ± 0.12d | - | - | - |
| **5** | α-Terpineol | 1,192 | 1,189 | 0.95 ± 0.01f | - | 16.84 ± 1.72d | 20.34 ± 0.85c | - | - | - | 11.25 ± 1.68e | 29.45 ± 1.85a | - | 20.72 ± 0.03c | 23.98 ± 2.4b | - |
| **6** | α-Terpineol acetate | 1,356 | 1,350 | - | - | 75.72 ± 1.84b | 71.58 ± 0.86c | - | - | - | 80.44 ± 1.40a | 60.21 ± 1.30e | - | 72.17 ± 0.16c | 67.11 ± 2.38d | - |
| **7** | Sabinen | 972 | 974 | 0.81 ± 0.01d | - | 0.94 ± 0.04c | 1.05 ± 0.05b | - | - | - | 0.99 ± 0.07bc | 1.05 ± 0.08b | - | 0.42 ± 0.04e | 1.14 ± 0.11a | - |
| **8** | α-Thujene | 926 | 929 | 0.91 ± 0.01bc | 2.09 ± 0.02a | - | - | 1.22 ± 0.13b | 0.48 ± 0.01cd | - | - |  | 0.52 ± 0.05c | - | - | 0.75 ± 0.15c |
| **9** | 1R-α-Pinene | 932 | 929 | 1.60 ± 0.01a | 1.04 ± 0.01b | - | - | 0.72 ± 0.09c | - | - | - | - | - | - | - | - |
| **10** | β-Myrcene | 991 | 991 | 0.96 ± 0.01bc | 1.73 ± 0.01a | - | 0.80 ± 0.03cd | 1.25 ± 0.14b | 0.49 ± 0.03d | - | - | 0.80 ± 0.03cd | 1.05 ± 0.27bc |  | 0.82 ± 0.05cd | 0.66 ± 0.07cd |

**Continued Supplementary Table S6**

| **No.** | **VOC** | **RI Cal^a^** | **RI Lit^b^** | **Relative content (%)^c^** | | | | | | | | | | | | |
| --- | --- | --- | --- | --- | --- | --- | --- | --- | --- | --- | --- | --- | --- | --- | --- | --- |
|  |  |  |  | **Tq** | **Tve** | **F_1_-1** | **F_1_-2** | **F_1_-3** | **F_1_-4** | **F_1_-5** | **F_1_-6** | **F_1_-7** | **F_1_-8** | **F_1_-9** | **F_1_-10** | **F_1_-11** |
| **11** | 3-Octanone | 987 | 986 | 5.84 ± 0.28a | 2.57 ± 0.01c | - | - | 0.84 ± 0.15g | 2.06 ± 0.10d | 1.88 ± 0.41de | 1.63 ± 0.38de | 1.52 ± 0.23ef | 2.97 ± 0.13c | 0.86 ± 0.04g | 1.11 ± 0.02fg | 4.22 ± 0.58b |
| **12** | α-Terpinene | 1,016 | 1,017 | 1.64 ± 0.01c | 3.23 ± 0.03a | - | - | 2.42 ± 0.17b | 1.14 ± 0.15de | 1.50 ± 0.23cd | - | - | 1.71 ± 0.21c | - | - | 0.80± 0.09e |
| **13** | D-Limonene | 1,028 | 1,032 | 0.89 ± 0.01e | 0.90 ± 0.03e | 2.66 ± 0.31cd | 2.96 ± 0.18bc | 0.70 ± 0.08e | - | - | 3.28 ± 0.36ab | 2.47 ± 0.13d | - | 3.55 ± 0.03a | 3.19 ± 0.43b | - |
| **14** | Eucalyptol | 1,030 | 1,032 | 5.78 ± 0.01a | 0.81 ± 0.02cd | 1.09 ± 0.19c | 0.32 ± 0.31e | 0.90 ± 0.08c | 0.22 ± 0.08ef | - | 1.54 ± 0.15b | - | 0.94 ± 0.14c | 0.96 ± 0.04c | 0.60 ± 0.11d | - |
| **15** | β-Caryophyllene | 1,421 | 1,419 | 1.74 ± 0.01ef | 3.37 ± 0.04cd | 2.45 ± 0.30de | 2.01 ± 0.10ef | 3.36 ± 0.72cd | 12.31 ± 2.11a | 4.09 ± 0.12c | 1.11 ± 0.14f | 0.72 ± 0.12f | 1.48 ± 0.19ef | 1.04 ± 0.14f | 1.22 ± 0.04ef | 8.61 ± 0.81b |
| **16** | Camphene | 946 | 952 | 2.60 ± 0.01a | - | - | - | 0.63 ± 0.07b | - | - | - | - | - | - | - | - |
| **17** | Cis-Sabinene hydrate | 1,066 | 1,070 | 0.26 ± 0.02d | 0.46 ± 0.01c | - | - | 0.95 ± 0.09b | 0.83 ± 0.08b | 3.03 ± 0.34a | - | - | 0.87 ± 0.09b | - | - | - |
| **18** | Linalool | 1,100 | 1,099 | 1.17 ± 0.03d | 1.06 ± 0.02de | - | - | 2.20 ± 0.20b | 0.97 ± 0.03e | 3.35 ± 0.16a | - | - | - | - | 0.68 ± 0.11f | 1.49 ± 0.10c |
| **19** | Camphor | 1,143 | 1,145 | 4.39 ± 0.02a | - | - | - | 0.72 ± 0.06b | - | - | - | - | - | - | - | - |
| **20** | L-Borneol | 1,165 | 1,166 | 7.89 ± 0.06a | 0.55 ± 0.01c | - | - | 0.92 ± 0.04b | - | - | - | - | - | - | - | - |

**Continued Supplementary Table S6**

| **No.** | **VOC** | **RI Cal^a^** | **RI Lit^b^** | **Relative content (%)^c^** | | | | | | | | | | | | |
| --- | --- | --- | --- | --- | --- | --- | --- | --- | --- | --- | --- | --- | --- | --- | --- | --- |
|  |  |  |  | **Tq** | **Tve** | **F_1_-1** | **F_1_-2** | **F_1_-3** | **F_1_-4** | **F_1_-5** | **F_1_-6** | **F_1_-7** | **F_1_-8** | **F_1_-9** | **F_1_-10** | **F_1_-11** |
| **21** | Thymoquinone | 1,251 | 1,250 | - | - | - | - | 0.97 ± 0.03c | 1.25 ± 0.19c | 3.92 ± 0.74a | - | - | 2.49 ± 0.87b | - | - | - |
| **22** | Thymol methyl ether | 1,236 | 1,235 | - | 2.73 ± 0.04c | - | - | - | 9.77 ± 0.45a | - | - | - | 3.81 ± 0.28b | - | - | 9.06 ± 1.43a |
| **23** | Carvacrol methyl ether | 1,244 | 1,244 | - | - | - | - | - | 7.81 ± 0.38b | - | - | - | 5.92 ± 0.08c | - | - | 9.87 ± 1.48a |
| **24** | Germacrene D | 1,483 | 1,481 | 0.59 ± 0.01d | - | - | 0.05 ± 0.08e | 2.43 ± 0.65b | 1.91 ± 0.20c | - | - | 0.35 ± 0.02de | - | - | - | 3.08 ± 0.45a |

^a^ RI Cal, calculated according to C7–C40.

^b^ RI Lit, obtained by searching the mass spectrum database NIST v14.0.

^c^ Tq, *T. quinquecostatus*; Tve, *T.* *vulgaris* ‘Elsbeth’.
