# Supplementary material for: Creation of new germplasm resources, development of SSR markers, and screening of monoterpene synthases in thyme
Source: BMC Plant Biol. 2023 Jan 6;23:13. doi: 10.1186/s12870-022-04029-2 (PMC9817278; doi:10.1186/s12870-022-04029-2)
Supplement: Supplementary file 9 — Additional file 9: Supplementary Table S7. Reported amino acid sequences used to construct the TPS phylogenetic tree. [file 12870_2022_4029_MOESM9_ESM.docx]

**Supplementary Table S7 Reported amino acid sequences used to construct the TPS phylogenetic tree.**

| **TPS family** | **Gene name** | **Species** | **Amino acid sequence ID in NCBI** | **TPS function** |
| --- | --- | --- | --- | --- |
| TPS-a | *AtTPS11* | *Arabidopsis thaliana* | Q4KSH9 | α-barbatene synthase |
|  | *AtTPS12* | *Arabidopsis thaliana* | Q9T0J9 | (Z)-γ-bisabolene synthase |
|  | *AtTPS13* | *Arabidopsis thaliana* | Q9T0K1 | (Z)-γ-bisabolene synthase |
|  | *AtTPS21* | *Arabidopsis thaliana* | Q84UU4 | α-humulene/(-)-(E)-β-caryophyllene synthase |
|  | *CsFS* | *Cucumis sativus* | AAU05951 | E,E-α-farnesene synthase |
|  | *CoTPS2* | *Cananga odorata* | MN230106 | terpene synthase |
|  | *AaFS* | *Artemisia annua* | AAX39387 | (E)-β-farnesene synthase |
|  | *LeGCS* | *Solanum lycopersicum* | AAC39431 | epidermal germacrene C synthase |
|  | *LoTPS3* | *Lathyrus odoratus* | MT598647 | β-caryophyllene/α-humulene |
|  | *LoTPS8* | *Lathyrus odoratus* | MT598650 | cubebene synthase |
| TPS-b | *AtTPS03* | *Arabidopsis thaliana* | A4FVP2 | (E)-β-ocimene synthase |
|  | *AtTPS10* | *Arabidopsis thaliana* | Q9ZUH4 | (E)-β-ocimene synthase |
|  | *AtTPS27* | *Arabidopsis thaliana* | P0DI76 | 1,8-cineole synthase |
|  | *MdFS* | *Malus domestica* | AAS01424 | α-farnesene synthase |
|  | *PcFS* | *Pyrus communis* | ABC25002 | α-farnesene synthase |
|  | *LjOS* | *Lotus japonicus* | AAT86042 | E-β-ocimene synthase |
|  | *CoTPS1* | *Cananga odorata* | MN230105 | terpene synthase |
|  | *CoTPS3* | *Cananga odorata* | MN230107 | terpene synthase |
|  | *AtTPS02* | *Arabidopsis thaliana* | NP_193406 | terpene synthase |
|  | *LoTPS7* | *Lathyrus odoratus* | MT598649 | geraniol synthase |
|  | *DoGES1* | *Dendrobium officinale* | MT875214 | chloroplast geraniol synthase |
|  | *PdGES* | *Prunus dulcis* | QEE82241 | trans-geraniol synthase |
|  | *ObGES* | *Ocimum basilicum* | AY362553 | geraniol synthase |
|  | *VvTPS* | *Vitis vinifera* | AAS79352 | (-)-α-terpineol synthase |
|  | *LoTPS4* | *Lathyrus odoratus* | MT598648 | ocimene synthase |
|  | *SaBBS* | *Santalum album* | AIV42941 | β-bisabolene synthase |
|  | *TcTPS02.1* | *Thymus caespititius* | KC181098 | γ-terpinene synthase |
|  | *TcTPS02.2* | *Thymus caespititius* | KC691293 | γ-terpinene synthase |
|  | *TcTPS05.1* | *Thymus caespititius* | KC181096 | α-terpineol synthase |
|  | *TcTPS05.2* | *Thymus caespititius* | KC181095 | α-terpineol synthase |
|  | *TvTPS1* | *Thymus vulgaris* | ALB78115 | γ-terpinene synthase |
|  | *TvTPS2* | *Thymus vulgaris* | JQ957864 | γ-terpinene synthase |
|  | *SauBS* | *Santalum austrocaledonicum* | ADO87003 | β-bisabolene synthase |

**Continued Supplementary Table S7**

| **TPS family** | **Name** | **Species** | **Amino acid sequence ID in NCBI** | **TPS** |
| --- | --- | --- | --- | --- |
| TPS-c | *AtCPS* | *Arabidopsis thaliana* | Q38802 | ent-copalyl diphosphate synthase |
|  | *NtCPS2* | *Nicotiana tabacum* | G3CCC0 | 8-hydroxy-copalyl diphosphate synthase |
|  | *SdKPS* | *Salvia divinorum* | A0A1S5RW73 | (-)-kolavenyl diphosphate synthase |
|  | *SmCPS5* | *Salvia miltiorrhiza* | A0A0A7ANR2 | ent-copalyl diphosphate synthase |
| TPS-d | *PaFS* | *Picea abies* | AAS47697 | E,E-α-farnesene synthase |
|  | *PtFS* | *Picea taeda* | AAO61226 | α-farnesene synthase |
| TPS-e/f | *AtKS* | *Arabidopsis thaliana* | Q9SAK2 | ent-kaurene synthase |
|  | *NtABS* | *Nicotiana tabacum* | G3CCC1 | cis-abienol synthase |
|  | *SmKSL* | *Salvia miltiorrhiza* | C8XPS0 | miltiradiene synthase |
|  | *SmKSL2* | *Salvia miltiorrhiza* | A0A0A7ANA5 | ent-13-epi-manoyl oxide synthase |
|  | *AtTPS04* | *Arabidopsis thaliana* | NP_564772 | terpene synthase |
|  | *CbLIS* | *Clarkia breweri* | AAC49395 | S-linalool synthase |
|  | *LnTPS3* | *Laurus nobolis* | AKQ19359 | diterpene geranyllinalool synthase |
|  | *AdAFS1* | *Actinidia deliciosa* | ACO40485 | terpene synthase |
|  | *VvCSENerGI* | *Vitis vinifera* | NP_001268004 | P(E)-nerolidol/(E,E)-geranyl linalool synthase |
|  | *PtTPS10* | *Populus trichocarpa* | AII32474 | terpene synthase |
|  | *VvPNENerGI* | *Vitis vinifera* | NP_001268201 | (E,E)-geranyllinalool synthase-like |
|  | *CbLIS2* | *Clarkia breweri* | AAD19840 | linalool synthase |
| TPS-g | *PlTPS2* | *Phaseolus lunatus* | AGS83387 | terpene synthase |
|  | *MtTPS3* | *Medicago truncatula* | XP_013457143 | (3S,6E)-nerolidol synthase |
|  | *VvPNLNGl1* | *Vitis vinifera* | ADR74213 | (3S)-linalool/(E)-nerolidol /(E,E)-geranyl linalool synthase |
|  | *VvPNLNGl2* | *Vitis vinifera* | ADR74214 | (3S)-linalool/(E)-nerolidol /(E,E)-geranyl linalool synthase |
|  | *VvPNLNGl3* | *Vitis vinifera* | ADR74215 | (3S)-linalool/(E)-nerolidol /(E,E)-geranyl linalool synthase |
|  | *VvPNLNGl4* | *Vitis vinifera* | ADR74216 | (3S)-linalool/(E)-nerolidol /(E,E)-geranyl linalool synthase |
|  | *AtTPS14* | *Arabidopsis thaliana* | Q84UV0 | S-(+)-linalool synthase |
