# Supplementary material for: Creation of new germplasm resources, development of SSR markers, and screening of monoterpene synthases in thyme
Source: BMC Plant Biol. 2023 Jan 6;23:13. doi: 10.1186/s12870-022-04029-2 (PMC9817278; doi:10.1186/s12870-022-04029-2)
Supplement: Supplementary file 10 — Additional file 10: Supplementary Fig. S3. Alignment of the deduced amino acid sequence of Tq04G005190.1 with the reported amino acid sequence of geraniol synthase ObGES. [file 12870_2022_4029_MOESM10_ESM.docx]

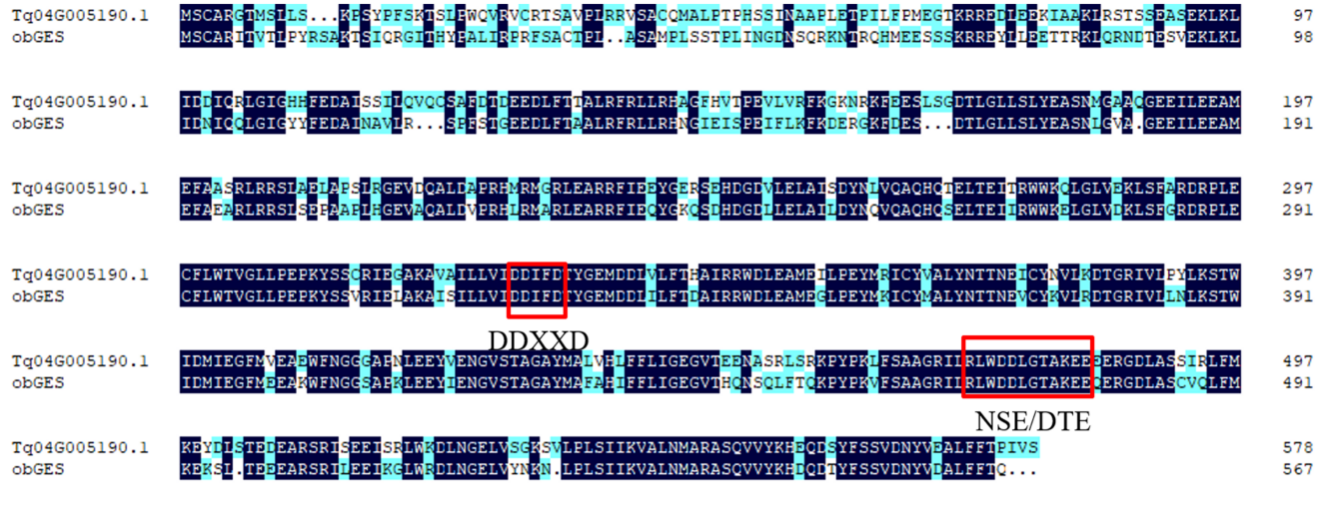


**Supplementary Fig. S3 Alignment of the deduced amino acid sequence of *Tq04G005190.1* with the reported amino acid sequence of geraniol synthase ObGES.** Red boxes indicate the conserved motifs (DDXXD and NSE/DTE) of TPSs.
