# Supplementary material for: Creation of new germplasm resources, development of SSR markers, and screening of monoterpene synthases in thyme
Source: BMC Plant Biol. 2023 Jan 6;23:13. doi: 10.1186/s12870-022-04029-2 (PMC9817278; doi:10.1186/s12870-022-04029-2)
Supplement: Supplementary file 11 — Additional file 11: Supplementary Table S8. Primer sequence, amplification length, amplification efficiency (%), and linear correlation coefficient (R2) of four TPS genes and two reference genes (18 S rRNA and β-actin). [file 12870_2022_4029_MOESM11_ESM.docx]

**Supplementary Table S8 Primer sequence, amplicon length, amplification efficiency (%), and linear correlation coefficient (R^2^) of four *TPS* genes and two reference genes (*18S rRNA* and *β-actin*).**

| **Gene** | **Primer sequence** | **Amplicon length (bp)** | **Amplification efficiency (%)** | **Linear correlation coefficient (R**2) |
| --- | --- | --- | --- | --- |
| ***18S rRNA*** | F：5'-AACGACTCTCGGCAACG-3' | 123 | 92.65 | 0.995 |
|  | R：5'-GCGTTCAAAGACTCGATG-3' |  |  |  |
| ***β-actin*** | F：5'-TGTGGATTGCCAAGGCAG-3' | 105 | 1.52 | 0.001 |
|  | R：5'-AATGAGCAGGCAGCAACA-3' |  |  |  |
| ***Tq13G005250.1*** | F：5'-ACGGATACCACAACATACTTTACCT-3' | 115 |  |  |
|  | R：5'-ATTGGACTGCTTTCGGCAC-3' |  |  |  |
| ***Tq02G0***02290.1 | F：5'-ATTTCGTCGAACTCCATACCG-3' | 118 |  |  |
|  | R：5'- TCCTTCCACGCTTCCCTAA-3' |  |  |  |
| ***Tq03G001560.1*** | F：5'-AGGAGGAGCGGTTGAATAGG-3' | 145 |  |  |
|  | R：5'-TCTCGTCCCGAAAGAAATAAGAC-3' |  |  |  |
| ***Tq04G005190.1*** | F：5'-AGGCGTTCGCTTGCTGA-3' | 225 |  |  |
|  | R：5'-TCGGTGAGTTCGGTCTGGT-3' |  |  |  |
